# A bisulfite-assisted and ligation-based qPCR amplification technology for locus-specific pseudouridine detection at base resolution

**DOI:** 10.1093/nar/gkae344

**Published:** 2024-05-06

**Authors:** Xin Fang, Ruiqi Zhao, Yafen Wang, Mei Sun, Jin Xu, Shengrong Long, Jing Mo, Hudan Liu, Xiang Li, Fang Wang, Xiang Zhou, Xiaocheng Weng

**Affiliations:** College of Chemistry and Molecular Sciences, Key Laboratory of Biomedical Polymers-Ministry of Education, Wuhan University, Wuhan, Hubei 430072, P. R. China; Department of Neurosurgery, Zhongnan Hospital of Wuhan University, Wuhan, Hubei 430071, P. R. China; School of Public Health, Wuhan University, Wuhan, Hubei 430071, P. R. China; College of Chemistry and Molecular Sciences, Key Laboratory of Biomedical Polymers-Ministry of Education, Wuhan University, Wuhan, Hubei 430072, P. R. China; Medical Research Institute, Wuhan University, Wuhan, Hubei 430071, P. R. China; Department of Neurosurgery, Zhongnan Hospital of Wuhan University, Wuhan, Hubei 430071, P. R. China; College of Chemistry and Molecular Sciences, Key Laboratory of Biomedical Polymers-Ministry of Education, Wuhan University, Wuhan, Hubei 430072, P. R. China; Medical Research Institute, Wuhan University, Wuhan, Hubei 430071, P. R. China; Department of Neurosurgery, Zhongnan Hospital of Wuhan University, Wuhan, Hubei 430071, P. R. China; Wuhan University School of Pharmaceutical Sciences, Wuhan 430071, China; College of Chemistry and Molecular Sciences, Key Laboratory of Biomedical Polymers-Ministry of Education, Wuhan University, Wuhan, Hubei 430072, P. R. China; Wuhan TaiKang Center for Life and Medical Sciences, Wuhan University, Wuhan, Hubei 430071, P. R. China; College of Chemistry and Molecular Sciences, Key Laboratory of Biomedical Polymers-Ministry of Education, Wuhan University, Wuhan, Hubei 430072, P. R. China; Wuhan TaiKang Center for Life and Medical Sciences, Wuhan University, Wuhan, Hubei 430071, P. R. China

## Abstract

Over 150 types of chemical modifications have been identified in RNA to date, with pseudouridine (Ψ) being one of the most prevalent modifications in RNA. Ψ plays vital roles in various biological processes, and precise, base-resolution detection methods are fundamental for deep analysis of its distribution and function. In this study, we introduced a novel base-resolution Ψ detection method named pseU-TRACE. pseU-TRACE relied on the fact that RNA containing Ψ underwent a base deletion after treatment of bisulfite (BS) during reverse transcription, which enabled efficient ligation of two probes complementary to the cDNA sequence on either side of the Ψ site and successful amplification in subsequent real-time quantitative PCR (qPCR), thereby achieving selective and accurate Ψ detection. Our method accurately and sensitively detected several known Ψ sites in 28S, 18S, 5.8S, and even mRNA. Moreover, pseU-TRACE could be employed to measure the Ψ fraction in RNA and explore the Ψ metabolism of different pseudouridine synthases (PUSs), providing valuable insights into the function of Ψ. Overall, pseU-TRACE represents a reliable, time-efficient and sensitive Ψ detection method.

## Graphical Abstract

**Graphical Abstract:**
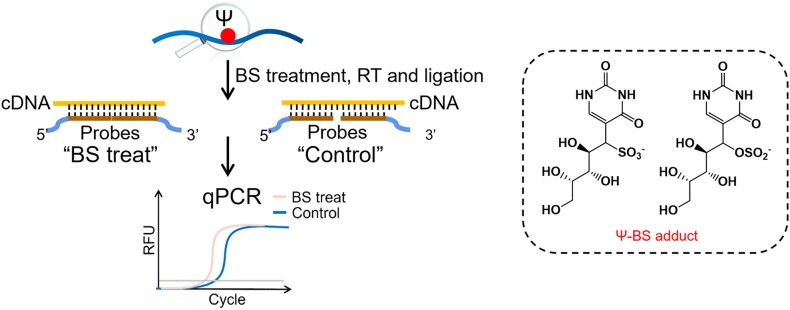

## Introduction

Post-transcriptional RNA modifications are common in RNA and play vital roles in various biological processes (1–3). Among these, Ψ, an isomer of uridine (U), is one of the most abundant modifications found in rRNA, tRNA and mRNA (4–6). Thirteen PUSs have been reported in the human genome (7), which function via two distinct mechanisms: RNA-dependent box H/ACA ribonucleoproteins and RNA-independent PUSs (8–11). These diverse PUSs are responsible for site-specific Ψ deposition in RNA. Numerous studies have revealed that Ψ could impact various cellular activities, such as RNA splicing, RNA stability, and translation (12–15). Notably, recent research indicated that Ψ in mRNA could enhance mRNA stability, and that the presence of Ψ-modified stop codons in mammalian mRNA could induce stop codon readthrough *in vivo* (16). Additionally, aberrant pseudouridylation has been linked with various human diseases, including T-acute lymphoblastic leukemia (T-ALL) (17), colorectal cancer (18), and glioblastoma (19).

Numerous methods have been developed to map Ψ sites in RNA. Previously, Ψ profiling methods primarily relied on the selective labeling of Ψ using N-cyclohexyl-N′-(2-morpholinoethyl)-carbodiimide metho-*p*-toluenesulfonate (CMC) to generate a CMC-Ψ adduct, resulting in termination of reverse transcription at one nucleotide 3′ to the CMC-modified Ψ site. By combining the CMC selective labeling strategy with high-throughput sequencing, techniques such as Ψ-seq, PSI-seq, and Pseudo-seq were developed to enable transcriptome-wide profiling of Ψ at single-base resolution (20–22). Subsequently, an azide-modified CMC (N_3_-CMC) was introduced to enrich CMC-labeled Ψ-containing RNA, enabling the detection of low-abundance Ψ sites (CeU-seq) (23). In addition, the use of hydrazine and aniline to cleave RNA at U sites other than at adenosine (A), cytidine (C), guanosine (G), and Ψ sites led to the development of HydraPsiSeq, facilitating quantitative detection of Ψ (24). Recently, techniques like BID-seq and PRAISE were reported, which used selective Ψ labeling with BS for quantitative profiling of Ψ across the whole transcriptome (16,25). BS could selectively react with Ψ under specific conditions to quantitatively generate Ψ-BS adduct without cytosine deamination. The resulting Ψ-BS adduct introduced a deletion signature at Ψ sites during reverse transcription (26), thus realizing quantitative detection of Ψ in combination with high-throughput sequencing. These studies have not only revealed plenty of Ψ sites in RNA but have also shed light on the biological function of Ψ.

Although high-throughput sequencing methods provide valuable transcriptome-wide information on Ψ, they are time-consuming and require sophisticated bioinformatics analysis. When investigating specific Ψ sites in individual RNA of interest, locus-specific Ψ detection methods are crucial. Various methods have been developed for the detection of locus-specific RNA modifications (27–31). For instance, ‘SELECT’ has been widely employed for m^6^A locus detection, which integrated an elongation and ligation technique with qPCR amplification method (32). Furthermore, a locus-specific Ψ detection method has also been developed using a CMC labeling strategy, which could induce mutation, deletion or termination during reverse transcription, allowing Ψ detection through the analysis of melting temperatures or amplification signals of qPCR products (16,33,34). However, the Ψ labeling efficiency with CMC is not satisfactory, potentially affecting the sensitivity and accuracy of quantification. Therefore, more efficient methods for locus-specific Ψ detection are highly desirable. Herein, we presented a robust method for locus-specific Ψ detection at base resolution, basing on ligation-assisted qPCR amplification technology (pseU-TRACE). This method used BS to selectively label Ψ, which would induce a unique deletion signature at Ψ sites during reverse transcription, subsequently altering the nick ligation efficiency of two complementary DNA probes, enabling site-specific Ψ detection with qPCR at base resolution.

## Materials and methods

### Materials

RNAs used in this work were purchased from Takara, the sequences were listed in Supplementary Table S1. siRNA and si-NC were purchased from GenePharma, the sequences were listed in Supplementary Table S3. DNA used in this work were purchased from Genecreate, the sequences were listed in Supplementary Tables S2, S4, S5 and S6.

The TRUB1, PUS7, Tubulin and GAPDH proteins were detected using the antibodies anti-TRUB1 (Proteintech, 12520-1-AP, 1:1000), anti-PUS7 (Abcam, ab289857, 1:1000), anti-Tubulin (Abclonal, 1:5000) and anti-GAPDH (Abclonal, 1:50 000). Primary murine T-ALL cells and healthy murine thymocytes were gifts from Prof. Hudan Liu.

### Cell culture and RNA extraction

HEK293T and HeLa cells were maintained in Dulbecco's modified eagle's medium (DMEM) (high glucose) supplemented with 10% fetal bovine serum (FBS), and 1% penicillin/streptomycin (Beijing Ding-guo changsheng Biotechnology Co., Ltd, GA3502) at 37°C with 5% CO_2_. Total RNA was extracted using TRIzol reagent (Invitrogen, 15596018) according to the manufacturer's instructions. PolyA-RNA was isolated from total RNA through two rounds of poly(A)+ selection with Oligo(dT)_25_ magnetic beads (NEB, S1419S).

### siRNA transfection

Cells were transfected with siRNA or si-NC using lipofectamine RNAiMAX transfection reagent (Invitrogen, 13778075) following the commercial protocols. We collected samples at different time point: 24 and 48 h for RNA analysis, and 48 and 72 h for protein analysis. Knockdown efficiency was assessed by qPCR for mRNA level test and western blot for protein level test. The siRNA sequences were listed in Supplementary Table S3 and the qPCR primers could be found in Supplementary Table S4.

### HPLC and ESI-MS analysis

Synthetic 19-Ψ was incubated with BS or CMC, respectively. The treatment procedures of 19-RNA with BS were shown in the following context. 19-RNA was reacted with 0.2 M CMC in BEU buffer (7 M urea, 4 mM EDTA pH 8.0, 50 mM Bicine pH 8.5) at 37°C for 20 min followed by purification with ethanol precipitation. Purified RNA was mixed with sodium carbonate buffer (100 mM Na_2_CO_3_ pH 10.4) in a 1:1 ratio and incubated at 37°C for 3 h to remove CMC from U and G residues. RNA was purified by ethanol precipitation. The raw material and the products after reaction with BS or CMC were passed into Dionex Ultimate 3000 hybrid LTQ Orbitrap Elite Velos Pro (Thermo Scientific) with a Thermo Scientifific Hypersil ODS Column (250 mm × 4.6 mm, 5 μm, C18). The gradient of elution buffer for HPLC is shown below (for BS: solvent A: 20 mM NH_4_AC buffer pH 7.0, solvent B: methanol, the procedures was shown in Table 1; for CMC: solvent A: 50 mM TEAA buffer pH 7.0, solvent B: acetonitrile, the procedures was shown in Table 2):

**Table 1.** The procedures used for the HPLC analysis of the reaction between Ψ-containing RNA and BS

Time (min)	Flow (ml min^−1^)	%A	%B
0	1	95	5
5	1	95	5
40	1	60	40
45	1	0	100
50	1	0	100
51	1	95	5

**Table 2.** The procedures used for the HPLC analysis of the reaction between Ψ-containing RNA and CMC

Time (min)	Flow (ml min^−1^)	%A	%B
0	1	90	10
5	1	90	10
45	1	75	25
50	1	0	100
55	1	0	100
56	1	90	10

Then the samples with different retention times were recovered separately, and the recovered samples were analyzed by ESI-MS. The ESI-MS recorded the signals using negative reflector mode.

### Western blot

HeLa cells were plated in 24-well plates and transfected with 10 pmol siRNA or si-NC. After 48 and 72 h of transfection, cells were washed once with PBS and lysed in 80 μl of 1 × SDS loading buffer (50 mm Tris–HCl pH 6.8, 10% glycerol, 2% SDS, 0.1% bromophenol blue, 1% beta-mercaptoethanol) at 70 rpm at 37°C for 10 min. The cell lysate was collected and then boiled at 95°C for 15 min. The appropriate amount of protein was loaded onto SDS-PAGE gels. The separated proteins were transferred onto a PVDF membrane (Millipore) in an ice-bath for 2 h. Then the PVDF membrane was blocked in 5% (w/v) BSA (Beijing Dingguo changsheng Biotechnology Co., Ltd, FA016) in TBST (Tris-buffered saline, 0.1% Tween 20) at 70 rpm at 37°C for 1 h. The blot of protein was stained with the corresponding antibody for at least 12 h at 4°C. The membrane was washed five times with TBST at 70 rpm at 37°C for 10 min each, then incubated with 1:5000 HRP-conjugated Affinipure Goat Anti-Rabbit IgG (H + L) (Proteintech, SA00001-2) or HRP-conjugated Affinipure Goat Anti-Mouse IgG (H + L) (Proteintech, SA00001-1) in 5% BSA (w/v) in TBST for 1 h at 70 rpm at 37°C. The membrane was washed five times with TBST as before and imaged on Molecular Imager ChemiDocTM XRS+ Imaging System (Bio-Rad) after incubation with Rhea ECL (US Everbright, Inc.).

### BS treatment and reverse transcription

RNA was subjected to bisulfite treatment according to the operations of BID-seq (16). 500 ng of RNA was mixed with 45 μl of freshly prepared BS reagent (2.4 M Na_2_SO_3_ and 0.36 M NaHSO_3_, prepared by dissolving 270 mg Na_2_SO_3_ and 34 mg NaHSO_3_ in 900 μl RNase-free H_2_O) and incubated at 70°C for 3 h. Then, 75 μl RNase-free H_2_O, 270 μl RNA binding buffer (RNA Clean and Concentrator-5 Kit, Zymo Research, R1016), and 400 μl ethanol were added to the reaction mixture, which was mixed well and loaded on an RNA Clean and Concentrator-5 collumn. After spinning and washing once with 200 μl RNA wash buffer (RNA Clean and Concentrator-5 Kit, Zymo Research, R1016), 200 μl RNA desulphonation buffer (Zymo Research, R5001-3-40) was added to the column and incubated at room temperature for 1 h. This was followed by spinning and washing twice with 700 μl RNA wash buffer, followed by eluting RNA with 10 μl RNase-free H_2_O. The BS treated RNA was labeled as ‘BS treat’ sample, while the BS untreated RNA was labeled as ‘Control’ sample. Both ‘BS treat’ and ‘Control’ were mixed with 1.0 μl 5 μM RT primer (1:1 mix of reverse transcription primers for the corresponding target RNA and GAPDH reverse transcription primers, the sequences were seen in Supplementary Table S5) at 65°C for 5 min and immediately moved onto ice. To this mixture, 4 μl 5× SSIV buffer, 2 μl 10 mM dNTP solution mix (NEB, N0447V), 1 μl 100 mM dithiothreitol (DTT), 0.5 μl Ribolock RNase Inhibitor (Thermo Scientific, EO0384) and 1 μl SuperScript IV reverse transcriptase (SSIV, Thermo Scientific, 18090050) were added. The reaction was mixed well and incubated at 50°C for 1 h, followed by adding 1 μl RNase H (NEB, M0297L) and incubating at 37°C for 20 min. The reaction mixture was heated at 70°C for 5 min and then the cDNA was purified using DNA Clean and Concentrator-5 Kit (Zymo Research, D4004). The eluted cDNAs (20 μl) were stored at −20°C.

### Ligation-based qPCR method

The ligation experiment was conducted based on the ‘SELECT’ method (32).1 μl cDNA from the previous step was mixed with 1 μl 0.01 μM up primer, 1 μl 0.01 μM down primer, 1 μl 10 × SplintR ligase reaction buffer (500 mM Tris–HCl, 100 mM MgCl_2_, 10 mM ATP, 100 mM DTT, pH 7.5) and 3 μl RNase-free H_2_O. The sequences of the probe primers were listed in Supplementary Table S6. The cDNA and primers were annealed by incubating the mixture at a temperature gradient: 90°C for 1 min, 80°C for 1 min, 70°C for 1 min, 60°C for 1 min, 50°C for 1 min, and then 40°C for 6 min. Subsequently, 2 μl 1 U SplintR ligase (NEB, M0375L) was added to the former mixture. The final reaction mixture was incubated at 40°C for 60 min, denatured at 95°C for 5 min and kept at 12°C. The reacted system was diluted directly with 40 μl RNase-free H_2_O. Afterwards, quantitative real-time PCR (qPCR) reaction was performed in a CFX-96 Real-Time System (Bio-Rad). All qPCR reactions were performed as 20 μl reactions using 2 × Hieff qPCR SYBR Green Master Mix (Yeasen), 1 μl 10 μM qPCR-FP, 1 μl 10 μM qPCR-RP and 2 μl finally diluted reaction mixture and ddH_2_O. qPCR was run under the following condition: 95°C for 5 min; (95°C for 10 s; 60°C for 35 s) × 40 cycles; 95°C for 15 s; 60°C for 1 min; 95°C for 15 s (collect fluorescence at a ramping rate of 0.05°C/s); 4°C hold. GAPDH was used as a housekeeping gene to measure the input and normalize the ‘BS treat’ and ‘Control’. The relative ligation products (relative amount) were calculated from the difference in C_T_ values between the ‘BS treat’ and ‘Control’. The relative ligation products were determined using 2^−ΔCT^. All assays were performed with at least two biological replicates and three technical replicates. The qPCR primers used in this study were listed in Supplementary Table S4. (FP stood for forward primer; RP stood for reverse primer). For mRNA expression testing, RNA was reverse transcribed to cDNA using the PrimeScript™ RT reagent Kit with gDNA Eraser (Perfect Real Time) (Takara, RR047A). qPCR reactions were performed as described above. The qPCR primers used in this study for mRNA expression testing were listed in Supplementary Table S4. Expression level was obtained by subtracting the housekeeping gene (GAPDH) C_T_ value from the target C_T_ value and normalizing to the non-targeting siRNA (si-NC).

### Ligation reaction conditions optimization

First, we optimized the ligation time of SplintR ligase. We selected 10, 20, 30, 45 and 60 min ligation times. Then, we optimized the ligation temperature: 37, 40 and 42°C. Finally, we performed ligase concentration gradient experiments at 40°C for 60 min, and selected 0, 0.1, 0.5, 1 and 2 U enzyme concentrations.

### The performance and detection limitation of pseU-TRACE

A set of different amounts of 60-Ψ (0.1, 1, 5, 10, 50, 100, 1000 fmol), 60-Ψ2 (0.001, 0.1, 1, 5,10, 50, 100, 1000 fmol), and 60-Ψ3 (1, 5,10, 50, 100, 1000 fmol) were subjected to BS treatment, reverse transcription, ligase ligation and qPCR amplification. The relative ligation products amounts of these oligos were calculated to assess the performance and detection limitation of pseU-TRACE.

### Determination of the Ψ fraction at specific site

First, 1 μg total RNA samples from HeLa and a set of different amounts of the standard RNA (2, 4, 6, 8, 10, 12, 16, 20, 30, 40 fmol) which shared the same sequence with a segment of 28S rRNA, were subjected to reverse transcription and qPCR. The C_T_ value of 2 fmol standard RNA was selected as the reference. Relative abundance of other samples with 2 fmol standard RNA was determined using 2^−ΔCT^. The relative abundance and the corresponding amounts were used as the fitting parameter to obtain a linear fit curve. Then, the amount of 28S rRNA in 1 μg total RNA samples were determined to be 6.34 fmol according to its relative abundance. Next, different ratio of standard RNA with a U or Ψ site were mixed to obtain total 6.34 fmol standard RNA mixture with different Ψ fractions, which were subjected to BS treatment, reverse transcription and qPCR. A linear plot was generated to quantify the Ψ fraction at the position 3938 of 28S rRNA in HeLa.

Determination of the Ψ fraction in a site of *SCP2* mRNA was performed in the same way. First, the amout of *SCP2* mRNA in 1 μg mRNA samples was determined to be 0.34 fmol using a newly synthetic standard RNA (0.02, 0.1, 0.2, 0.4, 0.6, 0.8, 1.2, 1.6, 2 fmol) that shared same sequence with a segment of *SCP2* mRNA. Then, 1 μg mRNA samples and 0.34 fmol standard RNA mixture with different Ψ fractions, were used to obtained a linear fit curve and calculated the Ψ fraction in *SCP2* mRNA. Further, the mRNA samples and standard RNA mixture were diluted. 500 ng mRNA smples and 0.17 fmol standard RNA mixture, were used to quantify the Ψ fraction in *SCP2* mRNA again.

## Results

### The development and principle of pseU-TRACE

**Figure 1.**
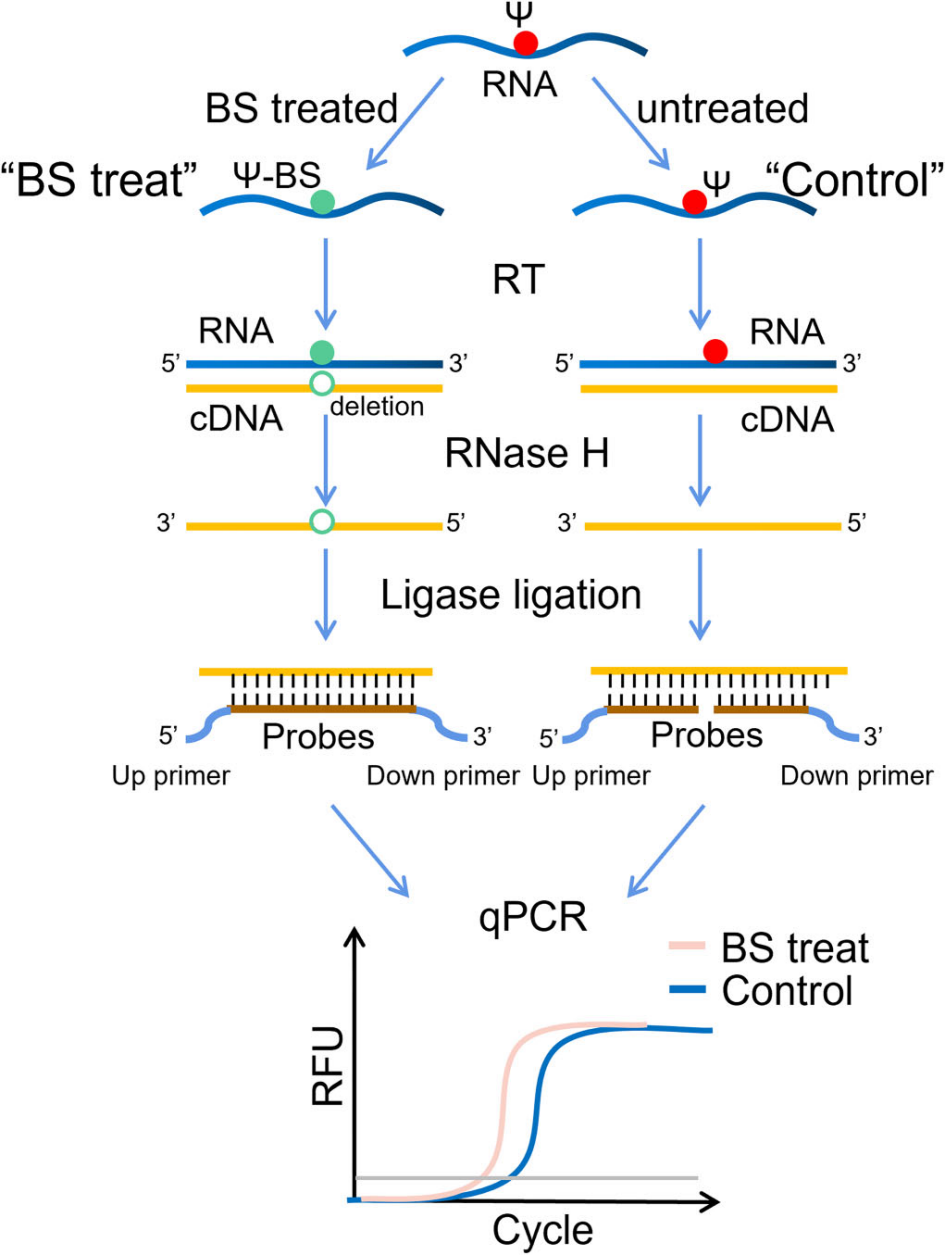
Schematic of pseU-TRACE. RNA containing Ψ was treated with BS, labeled as ‘BS treat’, while the BS-untreated sample was labeled as ‘Control’. ‘BS treat’ had a base deletion at Ψ sites during reserve transcription. And two probes were designed to complement the cDNA sequence on either side of the selected site. The two probes could be efficiently ligated by ligase in ‘BS treat’ due to the spatial proximity of the two probes, while ‘Control’ had a base gap which led to inefficient ligation. Locus-specific Ψ detection at base resolution could be achieved through qPCR amplification.

Based on previous advanced methods, we proposed a novel method for base-resolution Ψ detection, comprising three main steps: BS treatment, ligase ligation and qPCR amplification (Figure 1). BS could selectively react with Ψ under suitable conditions. The resulting Ψ-BS adduct induced a base deletion at Ψ sites during reverse transcription, leading to a base absence at Ψ sites in the cDNA of BS-treated RNA (referred to as ‘BS treat’). Two DNA probes were designed to complement the cDNA sequence on either side of the Ψ site position, with one probe having a phosphate modification at the 5′ end. These probes entirely complemented the cDNA of ‘BS treat’, resulting in enzymatic ligation by ligase and successful amplification in the subsequent qPCR, using the primer contained in the end of the probes. However, in the BS-untreated sample (referred to as ‘Control’), a base nick between the two probes inhibited ligase ligation and the subsequent qPCR amplification. Detection of Ψ was achieved by comparing the amounts of the ligation products between the two sets of samples (the relative ligation products or relative amount), which was determined by qPCR using 2^−ΔCT^.

### Feasibility verification of pseU-TRACE

Ψ detection could be achieved through both CMC labeling and BS labeling methods. While CMC labeling in combination with qPCR has been previously utilized for Ψ detection, its reaction efficiency with Ψ is relatively low (33). Therefore, there is a growing demand for more efficient methods for locus-specific Ψ detection. Previous studies have indicated that BS labeling is more efficient than CMC labeling (16,23). To validate this, we initially conducted HPLC and ESI-MS analysis experiments to compare the Ψ labeling efficiency of CMC and BS (Supplementary Figure S1). Our findings revealed that BS efficiently reacted with Ψ, almost entirely converting it to the Ψ-BS adduct under optimized conditions, while CMC’s reaction efficiency with Ψ was only about 60%, aligning with earlier reports. Subsequently, in order to assess the feasibility of our proposed pseU-TRACE method, we employed two single-stranded DNAs (59-T-G DNA and 60-T-G DNA) to simulate the post-reverse transcription products of RNA (Supplementary Table S2). We designed two probes (up primer and down primer) which complemented the DNA to assess the feasibility of SplintR ligase ligation (Supplementary Table S6). We observed that 59-T-G DNA, which was completely paired with both probes, produced significantly more ligation products than 60-T-G DNA, indicating that a one-base deletion significantly impacted the efficiency of SplintR ligase ligation (Supplementary Figure S2a). We then proceeded to optimize the conditions of ligation reaction, including time, temperature and concentration of ligase (Supplementary Figure S2b-d). A 60-minute reaction time, 40°C temperature, and 2 U enzyme concentration were ultimately selected, as these conditions yielded the highest ligation efficiency. Subsequently, we treated two 60-nucleotide (nt) RNAs containing either Ψ or U with BS, named 60-Ψ and YW-U (Supplementary Table S1). The sequences were different between the two oligos. After purification, the two RNAs were reverse transcribed and the template RNAs were degraded by RNase H. We then performed the ligation reaction and qPCR amplification. The results showed that Ψ-modified RNA could be accurately distinguished from U-containing RNA by analyzing the relative amount of ligation products in ‘BS treat’ and ‘Control’ (Supplementary Figure S2e). Notably, the relative amount in 60-Ψ reached up to 100-fold, indicating a significantly greater ligation products in ‘BS treat’ than ‘Control’, attributed to the base deletion at the Ψ site caused by BS treatment. However, no significant difference was noted in the ligation products of the YW-U sample, with or without BS treatment, as there was no base deletion in the cDNA after BS treatment. To further validate the base deletion at the Ψ site, we expanded the up primer with an A, T, C or G base for the ligation reaction. The results showed little ligation products were produced, confirming that the reverse transcription product of Ψ-containing RNA treated with BS indeed underwent a base deletion at Ψ sites, rather than a mismatch or other (Supplementary Figure S2f). Furthermore, to investigate the impact of surrounding sequences on the ligation site, we conducted ligation reactions and qPCR amplifications using various 59- or 60-nt DNAs with different nucleotides adjacent to the ligation site (Supplementary Tables S2 and S6). Using the relative ligation product amounts of T-G DNA as the reference, we observed that the sequence context influenced both ligase ligation and qPCR amplification efficiencies (Supplementary Figure S3a). Notably, samples with a 5′ C and a 3′ G adjacent to the ligation sites exhibited the highest efficiency, whereas those with both 5′ and 3′ A demonstrated the lowest. We also evaluated the performance and detection limits of the pseU-TRACE method. We selected three RNA strands with different sequences to explore the detection baseline: 60-Ψ (the nucleotides surrounding the Ψ site of RNA were 5′ C and 3′ A), 60-Ψ2 (the nucleotides surrounding the Ψ site of RNA were 5′ C and 3′ G), and 60-Ψ3 (the nucleotides surrounding the Ψ site of RNA were 5′ U and 3′ U). The cDNA sequence of these RNA oligos were corresponded to T-G, C-G and A-A cDNA in Supplementary Figure S3a, which showed middle, highest, and lowest ligation and qPCR amplification efficiency, respectively. Different amounts of these oligos were analyzed using pseU-TRACE. The results displayed that 60-Ψ2 RNA showed the lowest detection limitation, with the concentration of 0.01 fmol. (Supplementary Figure S3b–d). This result was consistent with previous results that the cDNA of 60-Ψ2 also showed the hightest ligation and qPCR amplification efficientcy. These resulsts suggested that different sequences may result in varying ligation and qPCR amplification efficiencies, and that the baseline for Ψ site detection could also differ among various sequences.

### Detection of Ψ in total RNA using pseU-TRACE

**Figure 2.**
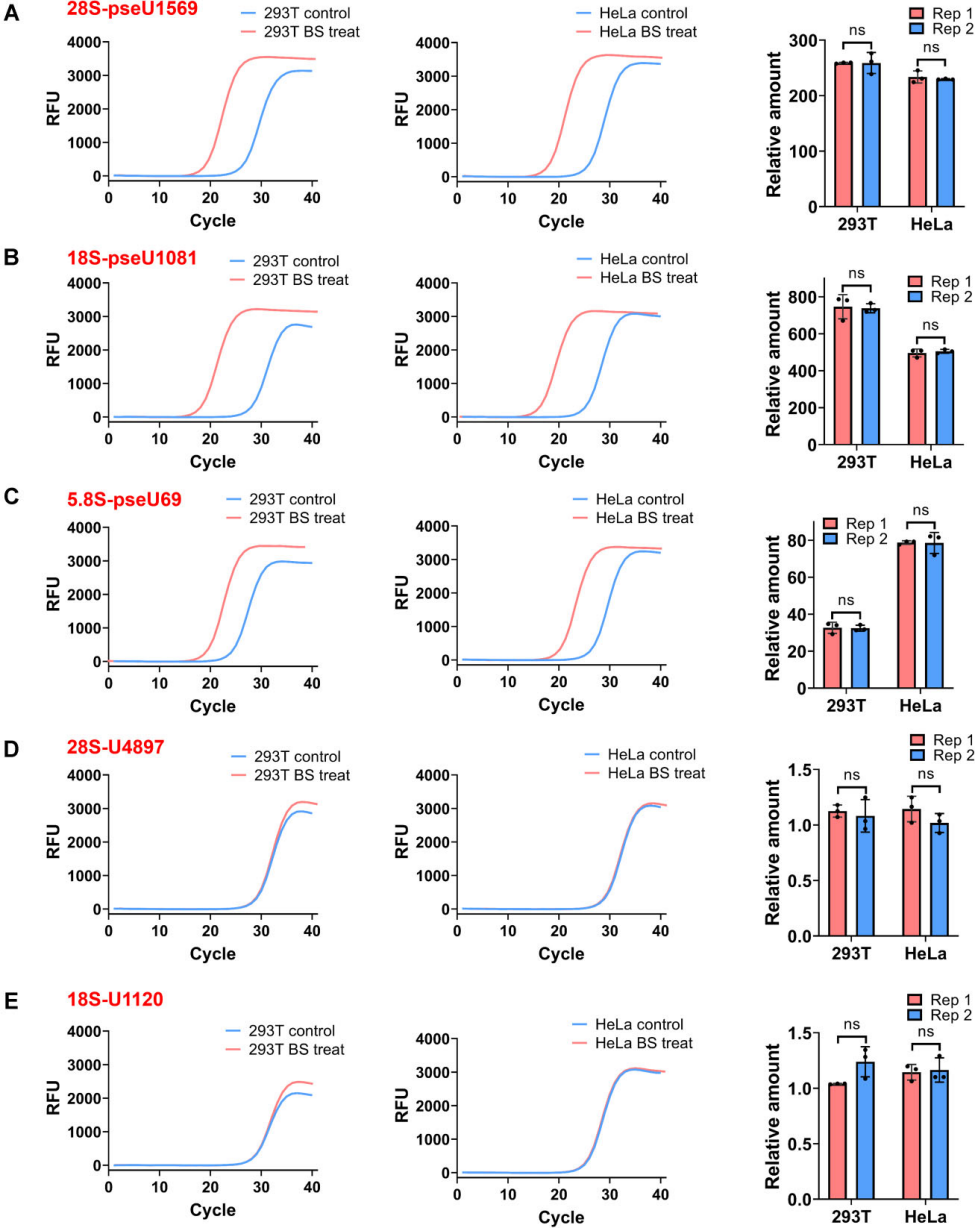
pseU-TRACE allowed the selective identification of Ψ sites in rRNA. (**A–C**) Real-time fluorescence amplification curves and calculated relative amount showed the pseU-TRACE results for Ψ detection in 28S, 18S and 5.8S. (**D, E**) Real-time fluorescence amplification curves and calculated relative amount displayed the pseU-TRACE results for U sites in 28S and 18S. The relative amount was determined using 2^−ΔCT^, where ΔC_T_ was calculated by the differences in the threshold cycle of amplification (C_T_ values) between ‘Control’ and ‘BS treat’. Specific Ψ sites were marked on the top left of the graphs. Each experiment was replicated twice (Rep1 and Rep2). Error bars indicate mean ± s.d. for three technical replicates. *P< 0.05; **P< 0.01; ***P< 0.001; ns, non-significant by *t*-test (one-tailed).

We then carried out pseU-TRACE for the validation of Ψ in total RNA. First, we chose to detect specific Ψ sites in 28S, 18S and 5.8S rRNA in HEK293T and HeLa cell lines (16,25). The results clearly revealed significant differences in the qPCR amplification curves (or C_T_ values) between the ‘BS treat’ and ‘Control’. Moreover, we observed that the ligation products of ‘BS treat’ were significantly higher than those in ‘Control’, with the observed highest relative amount of ligation products reaching 738-fold (Figure 2A–C, Supplementary Figure S4a–d). We observed that different sites displayed varying relative amounts of ligation products. We hypothesize this is primarily attributable to differences in the Ψ fractions at these sites. Additionally, the location of these sites within diverse sequences could influence the efficiency of ligase ligation and qPCR amplification. These results underscore the capability of pseU-TRACE for effective Ψ detection. Moreover, we noted that the C_T_ values of ‘Control’ were around 25–30, suggesting the presence of a small amount of ligation products. Despite a gap existing in the ‘Control’ samples, it appeared minimal ligation products that were produced by the ligase. However, in the samples without a gap (Ψ-containing RNA with BS treatment, referred to as ‘BS treat’), there was an increase in ligation products. Our approach for Ψ detection involved comparing the relative amounts of ligation products in these two scenarios. Moreover, to ensure pseU-TRACE could selectively detect Ψ, we examined the relative amount of ligation products at several other sites, including 28S-U4897, 28S-A1171, 28S-C2535, 28S-G920. The relative amount of ligation products at these sites was similar level, indicating that these sites were not labeled by BS and had no effect on the ligation reaction (Figure 2D and E, Supplementary Figure S4e–g). The results demonstrated the specificity of pseU-TRACE in detecting Ψ ranther than other base sites.

### Detection of Ψ in mRNA using pseU-TRACE

**Figure 3.**
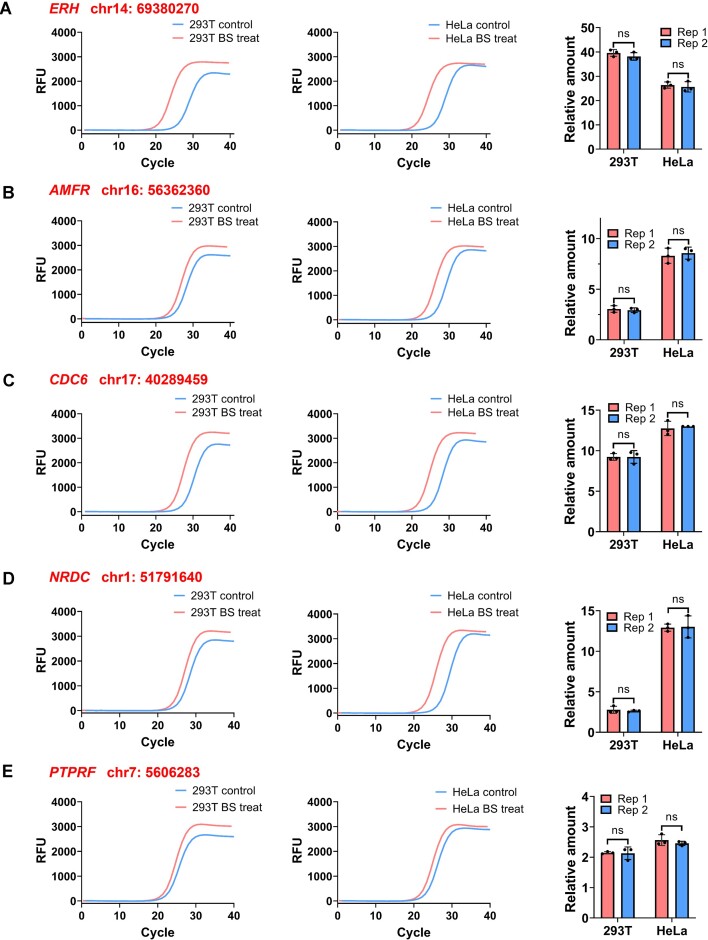
pseU-TRACE enabled the Ψ detection in mRNA. (**A–E**) Real-time fluorescence amplification curves and calculated relative amount showed the pseU-TRACE results of Ψ detection in mRNA. Specific Ψ sites were marked on the top and left of the graphs. Each experiment was replicated twice (Rep1 and Rep2). Error bars indicate mean ± s.d. for three technical replicates. *P< 0.05; **P< 0.01; ***P< 0.001; ns, non-significant by *t*-test (one-tailed).

We subsequently sought to detect several Ψ sites located in mRNA using total RNA samples (16,25). Impressively, there was at least a 3-fold distinction in the ligation products between ‘BS treat’ and ‘Control’, indicating that pseU-TRACE successfully detected these Ψ sites in *ERH*, *CDC6*, *DKC1* and *SCP2* mRNA from total RNA samples (Supplementary Figure S5). In the next step, we attempted to detect Ψ sites in mRNA after implementing a mRNA purification protocol. Starting with total RNA, we performed two consecutive rounds of poly(A)+ selection. Utilizing pseU-TRACE, we observed that the qPCR amplification curves (or C_T_ values) exhibited a clearer distinction between the ‘BS treat’ and ‘Control’ (Figure 3, Supplementary Figure S6). The relative amount of ligation products at the selected *ERH*, *CDC6* and *DKC1* Ψ sites all increased, with several sites displaying up to 40-fold distinction. These results confirmed the capability of pseU-TRACE to detect Ψ sites at base-resolution level. Collectively, these results demonstrated that pseU-TRACE was a highly effective method for the unequivocal identification of authentic Ψ sites in both rRNA and mRNA from biological samples.

### Ψ fraction determination using pseU-TRACE

**Figure 4.**
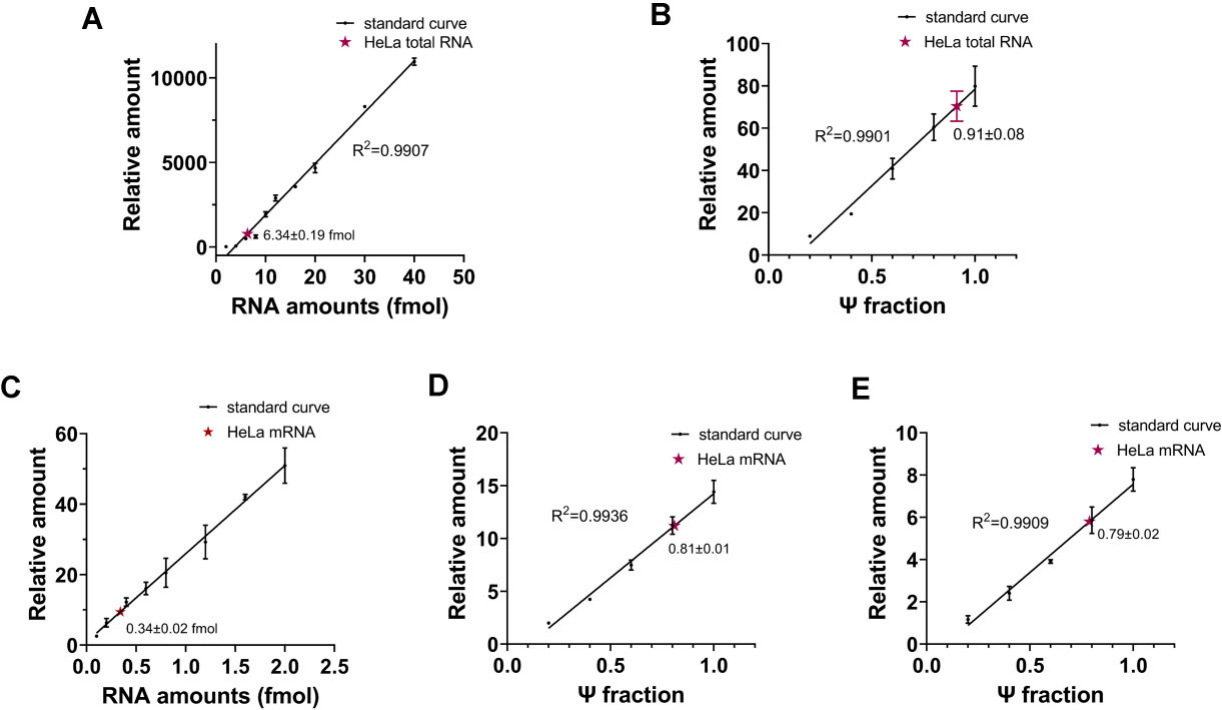
pseU-TRACE determined the Ψ fraction in 28S rRNA and mRNA in HeLa cells. (**A**) Quantification of 28S rRNA in 1 μg of HeLa total RNA. A series of different amounts of standard RNA containing a U at position 3938, along with 1 μg of HeLa total RNA, were subjected to reserve transcription and qPCR amplification. The relative amount was calculated by comparing the values obtained with the minimum amount. The amount of 28S calculated from the standard curve was 6.34 ± 0.19 fmol in 1 μg of HeLa total RNA. (**B**) Quantification of the Ψ fraction at position 3938 of 28S in HeLa. A series of 6.34 fmol standard RNA mixture with different Ψ fractions, achieved by mixing two synthetic 61 nt RNA oligonucleotides containing a Ψ or a U at position 3938, were subjected to pseU-TRACE analysis. The relative amount was calculated from the differences in C_T_ values between ‘BS treat’ and ‘Control’. (**C**) Quantification of *SCP2* mRNA in 1 μg of HeLa mRNA. The operations were the same as 28S rRNA. The amount of *SCP2* mRNA calculated from the standard curve was 0.34 ± 0.02 fmol in 1 μg of HeLa mRNA. (**D**) Quantification of the Ψ fraction in *SCP2* mRNA in 1 μg mRNA samples. The operations were the same as 28S rRNA. 1 μg mRNA samples and a series of 0.34 fmol standard RNA mixture with different Ψ fractions were subjected to pseU-TRACE analysis. (**E**) Quantification of the Ψ fraction in *SCP2* mRNA in 500 ng mRNA samples. RNA samples were diluted and performed pseU-TRACE analysis again in the same way. Error bars indicate mean ± s.d. for three technical replicates. Red star represented total RNA or mRNA samples from HeLa cells.

In addition to detecting cellular Ψ sites, pseU-TRACE also have the capability to quantify the Ψ fraction at specific Ψ sites. To verify this feasibility, we chose Ψ3938 in 28S rRNA as a proof of concept. Previous literature reported that the Ψ fraction at position 3938 in 28S rRNA approximated to 90% as determined by quantitative mass spectrometry (16). Initially, we quantified the cellular 28S rRNA amount. We used two synthetic 61 nt RNA oligonucleotides as standard RNA, one of which contained an internal U site at position 3938, while the other contained a Ψ site (the template sequence of 28S rRNA 3918–3978 nucleotides, Supplementary Table S1). Different amounts of the standard RNA with a U site at position 3938 were used for reverse transcription and qPCR, alongside total RNA samples from HeLa cells. We generated a fitted linear curve to quantify the amount of 28S rRNA (Figure 4A). Our results indicated that 1 μg of HeLa total RNA contained 6.34 ± 0.19 fmol of 28S. Subsequently, we used 6.34 fmol of standard RNA with various Ψ fractions (achieved by mixing U site- and Ψ site-containing RNA) to perform BS treatment, reverse transcription, ligation, and qPCR. A fitted linear curve was generated to quantify the Ψ fraction at position 3938 in 28S rRNA of HeLa cells (Figure 4B). The result showed that the Ψ fraction at position 3938 in HeLa cells was 0.91 ± 0.08, aligning closely with the quantitative results obtained from mass spectrometry in earlier studies.

Furthermore, we carried out Ψ fraction determination for *SCP2* mRNA, selecting a specific Ψ site within it. The operations were the same as 28S rRNA. Initially, we utilized two synthetic 61 nt RNA oligonucleotides as standard RNA (Supplementary Table S1) to determine the amount of *SCP2* mRNA present in HeLa cells (Figure 4C). The results indicated that 1 μg of HeLa mRNA contained approximately 0.34 ± 0.02 fmol of *SCP2* mRNA. Following this, we used 0.34 fmol of standard RNA with varying Ψ fractions, alongside 1 μg of HeLa mRNA, for BS treatment, reverse transcription, ligation, and qPCR. The resulting Ψ fraction in *SCP2* mRNA from HeLa cells was found to be 0.81 ± 0.01, which closely aligns with quantitative results from previous studies (Figure 4D) (16). We then investigated whether the quantity of input RNA would affect the accuracy of Ψ fraction quatification. The Ψ fraction of the same site was also determined using 500 ng input mRNA through diluting the standard RNA and 1μg of HeLa mRNA. Although the relative amount of standard RNA and 500 ng mRNA both decreased, the determination of Ψ fraction was 0.79 ± 0.02 (Figure 4E), which was similar to the quantitative result of 1 μg mRNA. These findings demonstrated that pseU-TRACE could not only allow for the precise validation of Ψ sites in rRNA and mRNA, but also enable the quantification of the Ψ fraction at specific Ψ sites.

### Application of pseU-TRACE

**Figure 5.**
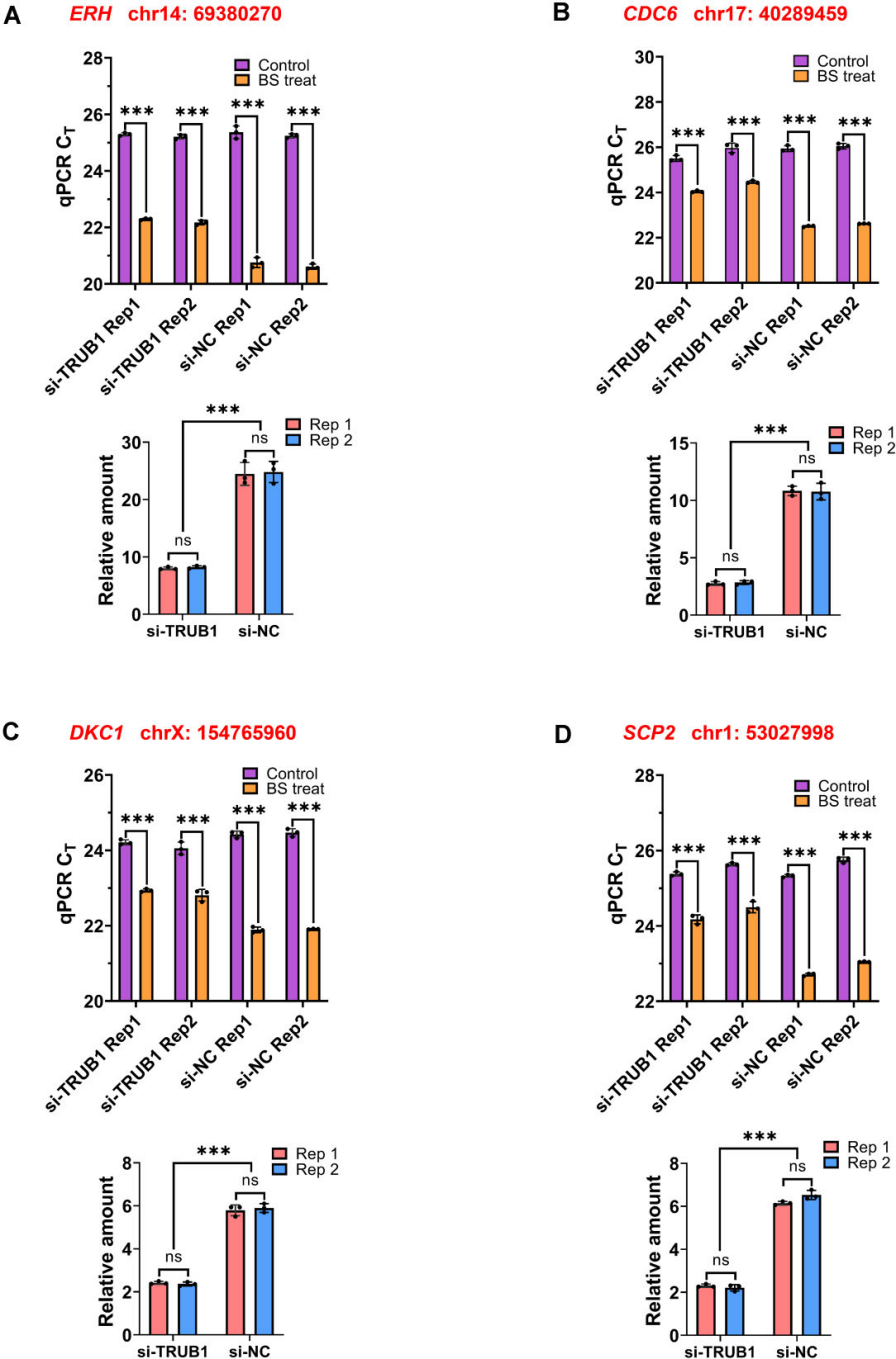
pseU-TRACE enabled functional studies of Ψ metabolism. pseU-TRACE combined with siRNA interference was used to identify the biological target sites of Ψ under TRUB1 regulation. (**A–D**) Real-time fluorescence amplification threshold cycle (C_T_ values) (top) and the calculated relative amount (bottom) showed the change of Ψ fractions in *ERH*, *CDC6*, *DKC1* and *SCP2* mRNA by comparing si-NC transfection with si-TRUB1 533 transfection. Specific Ψ sites were marked on the top and left of the graphs. Each experiment was replicated twice (Rep1 and Rep2). Error bars indicate mean ± s.d. for three technical replicates. *P< 0.05; **P< 0.01; ***P< 0.001; ns, non-significant by *t*-test (one-tailed).

As pseudouridylation is known to be catalyzed by thirteen PUSs, we aimed to determine whether pseU-TRACE could be used for functional studies of Ψ metabolism. For this purpose, we selected two pseudouridine synthases, TRUB1 and PUS7, along with several known Ψ sites under the regulation of the two enzymes as a proof-of-concept (16). We conducted transfection of small interfering RNA (siRNA) into HeLa cells and verified the efficiency of transfection by evaluating mRNA level through qPCR, and protein level through western blot (WB). A negative control was performed in parallel, where cells were transfected with a non-targeting siRNA termed si-NC. The results from both qPCR and WB showed successful reduction of TRUB1 and PUS7 expression (Supplementary Figure S7 and S8). We then selected *ERH*, *CDC6*, *DKC1*, *SCP2*, *NRDC* and *FSCN1* mRNA as targets for further investigation. We performed pseU-TRACE as previously described and observed that the relative amount of ligation products in samples transfected with the corresponding siRNA was significantly lower compared to samples transfected with si-NC. This reduction of ligation products indicated a decrease in the Ψ fractions, correlating to the knockdown of the related PUSs. It could be seen that Ψ sites in *ERH*, *CDC6*, *DKC1* and *SCP2* mRNA were primarily regulated by TRUB1, while those in *NRDC* and *FSCN1* were regulated by PUS7 (Figure 5, Supplementary Figure S9). These findings suggested that our strategy could achieve the functional studies of PUS regulation. We posit that our strategy will facilitate further understanding of Ψ regulation and metabolism.

Moreover, we conducted a preliminary investigation into the relationship between T-acute lymphoblastic leukemia (T-ALL) and Ψ. We learned that SHQ1, an H/ACA snoRNP assembly factor involved in snRNA pseudouridylation, was highly expressed in T-ALL, leading to a higher pseudouridylation at position Ψ54 in U2 snoRNA (17). We applied our pseU-TRACE in primary murine T-ALL cells and healthy murine thymocytes to explore whether there was a difference at Ψ54 in U2 snoRNA between the two types of cells. We discovered higher level of pseudouridylation at Ψ54 in U2 snoRNA in T-ALL cells compared to healthy cells (Supplementary Figure S10). This discovery not only demonstrated the feasibility of our approach, but also highlighted that the potential utility of pseU-TRACE to explore the connection between pseudouridylation and various diseases in future investigations.

## Discussion

In conclusion, we established a new strategy, pseU-TRACE, for the precise detection of Ψ in biological samples. This method involved a combination of BS treatment, ligase ligation and qPCR amplification, enabling the accurate identification of Ψ at base resolution in both rRNA and mRNA. In addition, pseU-TRACE could be utilized for quantifying the Ψ fraction in RNA, as well as for investigating the function of Ψ regulation. Characterized by its flexibility, accuracy, and selectivity, this approach holds potential for validating novel Ψ sites in future research. By integrating PUS interference with pseU-TRACE, we can conduct in-depth analyses of Ψ synthesis and metabolism, significantly advancing our understanding of Ψ’s functional roles.

## Supplementary Material

gkae344_Supplemental_File

## Data Availability

The data that support the findings of this study are available in the Supplementary Data of this article. Further information and requests for resources and reagents should be directed to and will be fulfilled by the lead contact, Xiaocheng Weng (xcweng@whu.edu.cn).

## References

[1] Frye M., Harada B.T., Behm M., He C. RNA modifications modulate gene expression during development. Science. 2018; 361:1346–1349. 30262497 10.1126/science.aau1646 PMC6436390

[2] Wang Y.F., Zhang X., Liu H., Zhou X. Chemical methods and advanced sequencing technologies for deciphering mRNA modifications. Chem. Soc. Rev. 2021; 50:13481–13497. 34792050 10.1039/d1cs00920f

[3] Boccaletto P., Machnicka M.A., Purta E., Piatkowski P., Baginski B., Wirecki T.K., de Crecy-Lagard V., Ross R., Limbach P.A., Kotter A. et al. MODOMICS: a database of RNA modification pathways. 2017 update. Nucleic Acids Res. 2018; 46:D303–D307. 29106616 10.1093/nar/gkx1030 PMC5753262

[4] Karijolich J., Yi C.Q., Yu Y.T. Transcriptome-wide dynamics of RNA pseudouridylation. Nat. Rev. Mol. Cell Biol. 2015; 16:581–585. 26285676 10.1038/nrm4040 PMC5694666

[5] Zhao Y., Dunker W., Yu Y.T., Karijolich J. The Role of Noncoding RNA Pseudouridylation in Nuclear Gene expression events. Front. Bioeng. Biotechnol. 2018; 6:8. 29473035 10.3389/fbioe.2018.00008 PMC5809436

[6] Li X.Y., Ma S.Q., Yi C.Q. Pseudouridine: the fifth RNA nucleotide with renewed interests. Curr. Opin. Chem. Biol. 2016; 33:108–116. 27348156 10.1016/j.cbpa.2016.06.014

[7] Hammal T., Ferre-D’Amare A.R. Pseudouridine synthases. Chem. Biol. 2006; 13:1125–1135. 17113994 10.1016/j.chembiol.2006.09.009

[8] Spenkuch F., Motorin Y., Helm M. Pseudouridine: still mysterious, but never a fake (uridine)!. RNA Biol. 2014; 11:1540–1554. 25616362 10.4161/15476286.2014.992278 PMC4615568

[9] Rintala-Dempsey A.C., Kothe U. Eukaryotic stand-alone pseudouridine synthases - RNA modifying enzymes and emerging regulators of gene expression?. RNA Biol. 2017; 14:1185–1196. 28045575 10.1080/15476286.2016.1276150 PMC5699540

[10] Kiss T., Fayet-Lebaron E., Jady B.E. Box H/ACA Small Ribonucleoproteins. Mol. Cell. 2010; 37:597–606. 20227365 10.1016/j.molcel.2010.01.032

[11] Carlile T.M., Martinez N.M., Schaening C., Su A., Bell T.A., Zinshteyn B., Gilbert W.V. mRNA structure determines modification by pseudouridine synthase 1. Nat. Chem. Biol. 2019; 15:966–974. 31477916 10.1038/s41589-019-0353-z PMC6764900

[12] Martinez N.M., Su A., Burns M.C., Nussbacher J.K., Schaening C., Sathe S., Yeo G.W., Gilbert W.V. Pseudouridine synthases modify human pre-mRNA co-transcriptionally and affect pre-mRNA processing. Mol. Cell. 2022; 82:645–659. 35051350 10.1016/j.molcel.2021.12.023 PMC8859966

[13] Yu A.T., Ge J.H., Yu Y.T. Pseudouridines in spliceosomal snRNAs. Protein & Cell. 2011; 2:712–725. 21976061 10.1007/s13238-011-1087-1 PMC4722041

[14] Cerneckis J., Cui Q., He C.A., Yi C.Q., Shi Y.H. Decoding pseudouridine: an emerging target for therapeutic development. Trends Pharmacol. Sci. 2022; 43:522–535. 35461717 10.1016/j.tips.2022.03.008

[15] Jack K., Bellodi C., Landry D.M., Niederer R.O., Meskauskas A., Musalgaonkar S., Kopmar N., Krasnykh O., Dean A.M., Thompson S.R. et al. rRNA pseudouridylation defects affect ribosomal ligand binding and translational fidelity from yeast to human cells. Mol. Cell. 2011; 44:660–666. 22099312 10.1016/j.molcel.2011.09.017 PMC3222873

[16] Dai Q., Zhang L.S., Sun H.L., Pajdzik K., Yang L., Ye C., Ju C.W., Liu S., Wang Y.R., Zheng Z. et al. Quantitative sequencing using BID-seq uncovers abundant pseudouridines in mammalian mRNA at base resolution. Nat. Biotechnol. 2023; 41:344–354. 36302989 10.1038/s41587-022-01505-w PMC10017504

[17] Su H.X., Hu J.C., Huang L., Yang Y., Thenoz M., Kuchmiy A., Hu Y.F., Li P., Feng H., Zhou Y. et al. SHQ1 regulation of RNA splicing is required for T-lymphoblastic leukemia cell survival. Nat. Commun. 2018; 9:4281. 30323192 10.1038/s41467-018-06523-4 PMC6189109

[18] Kan G.Y., Wang Z.Y., Sheng C.J., Chen G., Yao C., Mao Y.Z., Chen S. Dual inhibition of DKC1 and MEK1/2 synergistically restrains the growth of colorectal cancer cells. Adv. Sci. 2021; 8:2004344. 10.1002/advs.202004344 PMC8132060 34026451

[19] Cui Q., Yin K.L., Zhang X.T., Ye P., Chen X.W., Chao J.F., Meng H.W., Wei J.B., Roeth D., Li L. et al. Targeting PUS7 suppresses tRNA pseudouridylation and glioblastoma tumorigenesis. Nat. Cancer. 2021; 2:932–949. 35121864 10.1038/s43018-021-00238-0 PMC8809511

[20] Schwartz S., Bernstein D.A., Mumbach M.R., Jovanovic M., Herbst R.H., Leon-Ricardo B.X., Engreitz J.M., Guttman M., Satija R., Lander E.S. et al. Transcriptome-wide mapping reveals widespread dynamic-regulated pseudouridylation of ncRNA and mRNA. Cell. 2014; 159:148–162. 25219674 10.1016/j.cell.2014.08.028 PMC4180118

[21] Carlile T.M., Rojas-Duran M.F., Zinshteyn B., Shin H., Bartoli K.M., Gilbert W.V. Pseudouridine profiling reveals regulated mRNA pseudouridylation in yeast and human cells. Nature. 2014; 515:143–146. 25192136 10.1038/nature13802 PMC4224642

[22] Lovejoy A.F., Riordan D.P., Brown P.O. Transcriptome-wide mapping of pseudouridines: pseudouridine synthases modify specific mRNAs in *S. cerevisiae*. PLoS One. 2014; 9:e110799. 25353621 10.1371/journal.pone.0110799 PMC4212993

[23] Li X.Y., Zhu P., Ma S.Q., Song J.H., Bai J.Y., Sun F.F., Yi C.Q. Chemical pulldown reveals dynamic pseudouridylation of the mammalian transcriptome. Nat. Chem. Biol. 2015; 11:592–597. 26075521 10.1038/nchembio.1836

[24] Marchand V., Pichot F., Neybecker P., Ayadi L., Bourguignon-Igel V., Wacheul L., Lafontaine D.L.J., Pinzano A., Helm M., Motorin Y. HydraPsiSeq: a method for systematic and quantitative mapping of pseudouridines in RNA. Nucleic Acids Res. 2020; 48:e110. 32976574 10.1093/nar/gkaa769 PMC7641733

[25] Zhang M., Jiang Z., Ma Y., Liu W., Zhuang Y., Lu B., Li K., Peng J., Yi C. Quantitative profiling of pseudouridylation landscape in the human transcriptome. Nat. Chem. Biol. 2023; 19:1185–1195. 36997645 10.1038/s41589-023-01304-7

[26] Khoddami V., Yerra A., Mosbruger T.L., Fleming A.M., Burrows C.J., Cairns B.R. Transcriptome-wide profiling of multiple RNA modifications simultaneously at single-base resolution. Proc. Nat. Acad. Sci. U.S.A. 2019; 116:6784–6789. 10.1073/pnas.1817334116 PMC6452723 30872485

[27] Dai Q., Fong R., Saikia M., Stephenson D., Yu Y.T., Pan T., Piccirilli J.A. Identification of recognition residues for ligation-based detection and quantitation of pseudouridine and N^6^-methyladenosine. Nucleic Acids Res. 2007; 35:6322–6329. 17881375 10.1093/nar/gkm657 PMC2094055

[28] Liu N., Parisien M., Dai Q., Zheng G.Q., He C., Pan T. Probing N^6^-methyladenosine RNA modification status at single nucleotide resolution in mRNA and long noncoding RNA. RNA. 2013; 19:1848–1856. 24141618 10.1261/rna.041178.113 PMC3884656

[29] Harcourt E.M., Ehrenschwender T., Batista P.J., Chang H.Y., Kool E.T. Identification of a selective polymerase enables detection of N^6^-methyladenosine in RNA. J. Am. Chem. Soc. 2013; 135:19079–19082. 24328136 10.1021/ja4105792 PMC3905807

[30] Liu J.Z., Yang W., Zhang X., Wang Y.F., Zhou X. Bisulfite-free and quantitative detection of 5-formylcytosine in DNA through qPCR. Chem. Commun. 2021; 57:13796–13798. 10.1039/d1cc05987d 34877946

[31] Wang S.R., Wang J.Q., Zhang X.E., Fu B.S., Song Y.Y., Ma P., Gu K., Zhou X., Zhang X.L., Tian T. et al. N^6^-Methyladenine hinders RNA- and DNA-directed DNA synthesis: application in human rRNA methylation analysis of clinical specimens. Chem. Sci. 2016; 7:1440–1446. 29910902 10.1039/c5sc02902c PMC5975930

[32] Xiao Y., Wang Y., Tang Q., Wei L.H., Zhang X., Jia G.F. An elongation- and ligation-based qPCR amplification method for the radiolabeling-free detection of locus-specific N^6^-methyladenosine modification. Angew. Chem. Int. Ed. 2018; 57:15995–16000. 10.1002/anie.201807942 30345651

[33] Lei Z.X., Yi C.Q. A radiolabeling-free, qPCR-based method for locus-specific pseudouridine detection. Angew. Chem. Int. Ed. 2017; 56:14878–14882. 10.1002/anie.201708276 28960747

[34] Zhang W., Eckwahl M.H., Zhou K., Pan T. Sensitive and quantitative probing of pseudouridine modification in mRNA and long noncoding RNA. RNA. 2019; 25:1218–1225. 31227565 10.1261/rna.072124.119 PMC6800517

