# Supplementary material for: A bisulfite-assisted and ligation-based qPCR amplification technology for locus-specific pseudouridine detection at base resolution
Source: Nucleic Acids Res. 2024 May 6;52(10):e49. doi: 10.1093/nar/gkae344 (PMC11162771; doi:10.1093/nar/gkae344)
Supplement: gkae344_Supplemental_File [file gkae344_supplemental_file.pdf]

Supporting Information

A bisulfite-assisted and ligation-based qPCR amplification technology for locus-specific pseudouridine detection at base resolution

Xin Fang,†[1] Ruiqi Zhao,†[2] Yafen Wang,[3] Mei Sun,[1] Jin Xu,[4] Shengrong Long,[2] Jing Mo,[1] Hudan Liu,[4] Xiang Li,[2] Fang Wang,[6] Xiang Zhou,[1, 5] Xiaocheng Weng*[1, 5]

† These authors contributed equally to this work.

Supplementary Tables and Figures

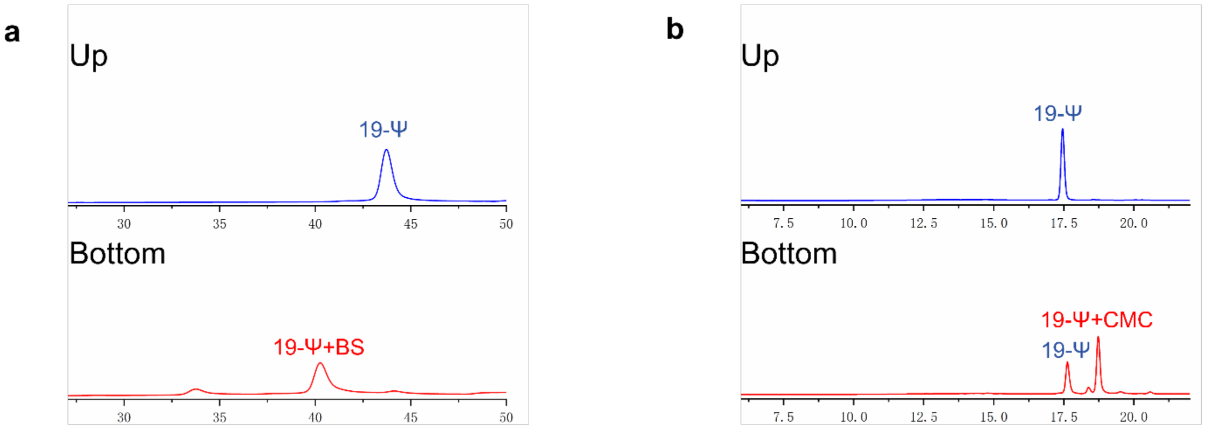

		Calculated	Found
Up	19-Ψ	6645.2	6645.6
Bottom	19-Ψ+BS	6727.2	6727.7

		Calculated	Found
Up	19-Ψ	6645.2	6646.1
Bottom	19-Ψ	6645.2	6646.0
Bottom	19-Ψ+CMC	6896.4	6897.2

Figure S1. Reaction efficiency of BS and CMC with Ψ. (a) HPLC analysis and ESI-MS analysis of 19 nt Ψ-containing RNA oligonucleotides untreated (Up) or treated with BS (bottom). (b) HPLC analysis and ESI-MS analysis of 19 nt Ψ-containing RNA oligonucleotides untreated (Up) or treated with CMC (bottom).

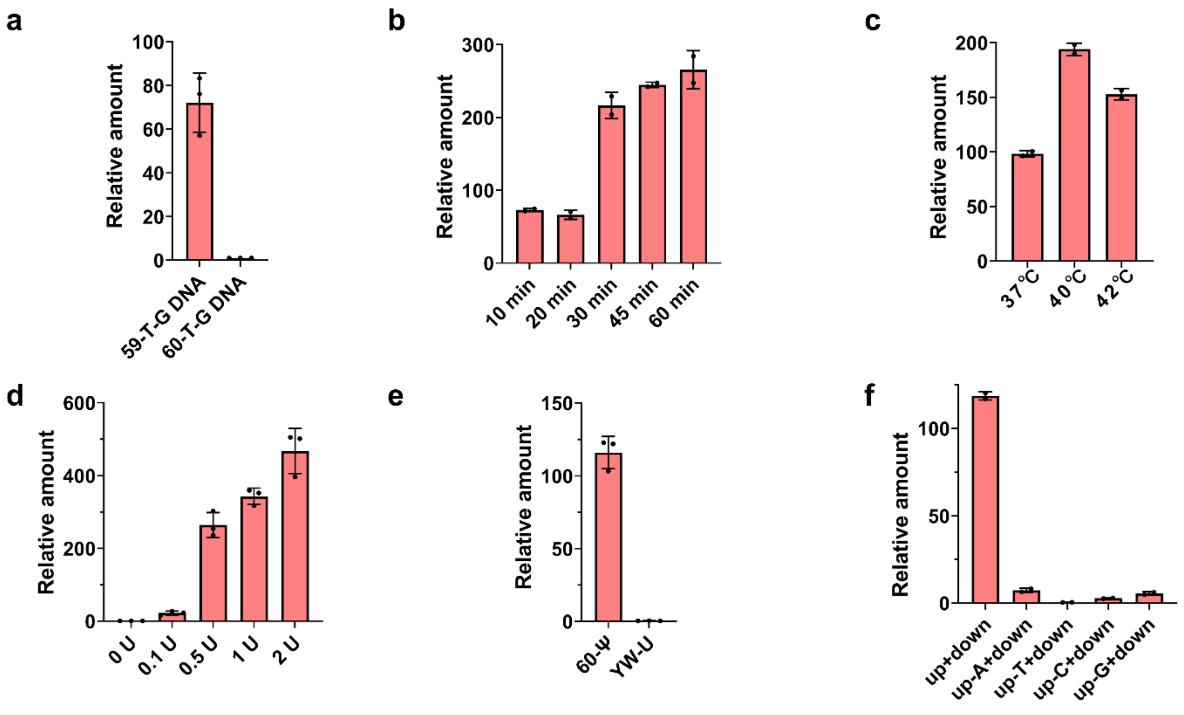

Figure S2. Preliminary experimental validation of pseU-TRACE. (a) Two single-stranded DNA and two probes complementary to the DNA were designed, and ligase ligation and qPCR amplification were performed. (b-d) Optimization of the ligation reaction time (b), ligation temperature (c), and ligase concentration (d). (e) 60 nt RNA containing Ψ or U was subjected to pseU-TRACE analysis. (f) Different up probes were designed to investigate whether BS treatment of Ψ does result in one base deletion rather than a mismatch. The relative amount was determined using $2^{-\Delta C_T}$. ΔC_T was calculated by the differences in the threshold cycle of amplification (C_T values) between "Control" and "BS treat". Error bars indicated mean±s.d. for 2 or 3 technical replicates. *p<0.05; **p<0.01; ***p<0.001; ns, non-significant by t-test (one-tailed).

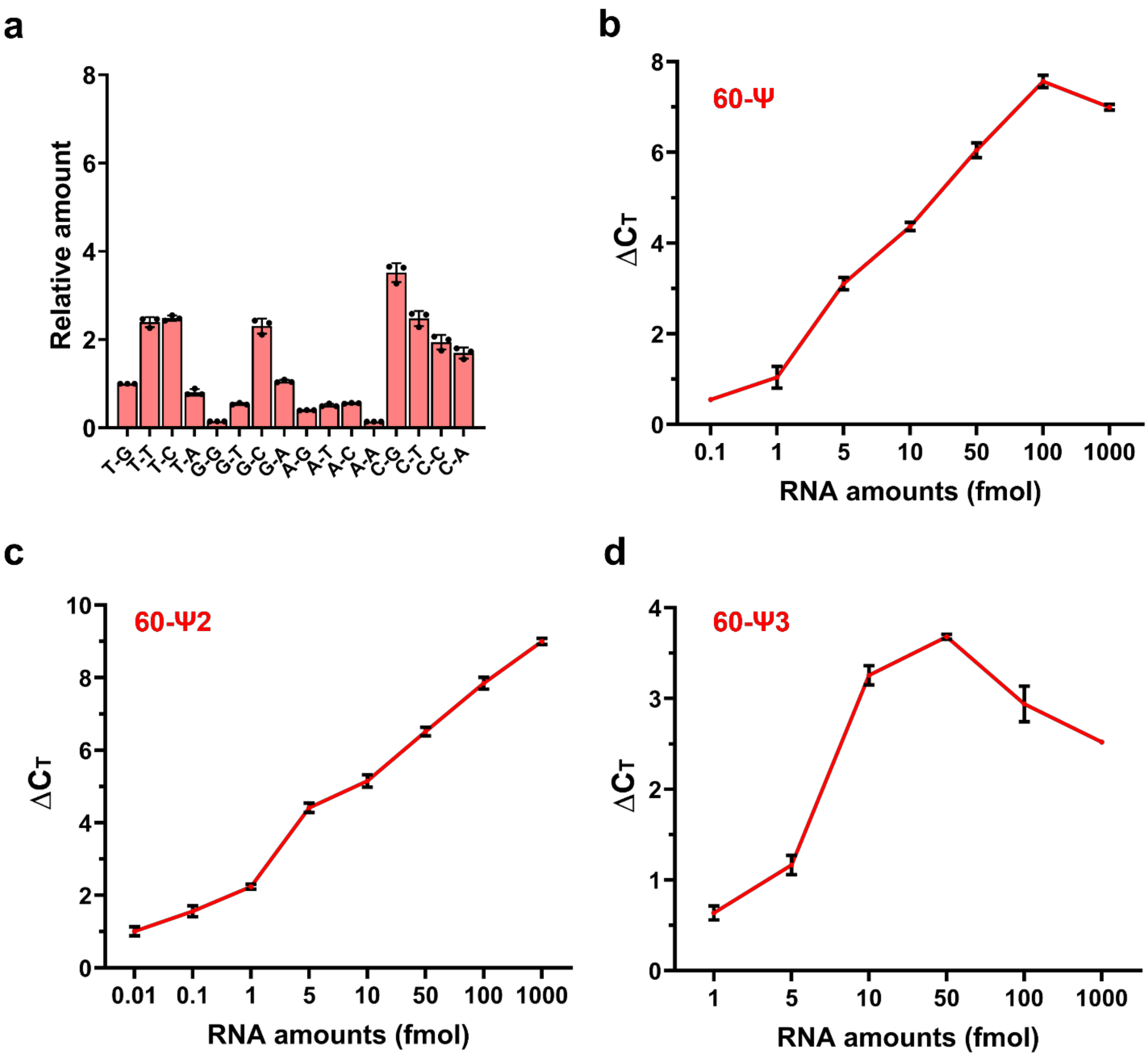

Figure S3. The performance and detection limits of pseU-TRACE. (a) A set of 59- or 60-nt DNA templates with different nucleotides beside the ligation site were conducted pseU-TRACE analysis to determine the influences of sequences to the ligation efficiency. The relative amount of ligation products of T-G DNA was used as the reference. (b-d) Different amounts of input RNA were performed pseU-TRACE to determine the baseline. The nucleotides surrounding the Ψ site of RNA were 5' C and 3' A (60-Ψ, b), 5' C and 3' G (60-Ψ2, c), 5' U and 3' U (60-Ψ3, d), respectively. And the cDNA sequence of these RNA oligos were corresponded to T-G, C-G and A-A cDNA in Figure S3 a. ΔC_T was calculated by the differences in the threshold cycle of amplification (C_T values) between "Control" and "BS treat". Error bars indicated mean±s.d. for 3 technical replicates.

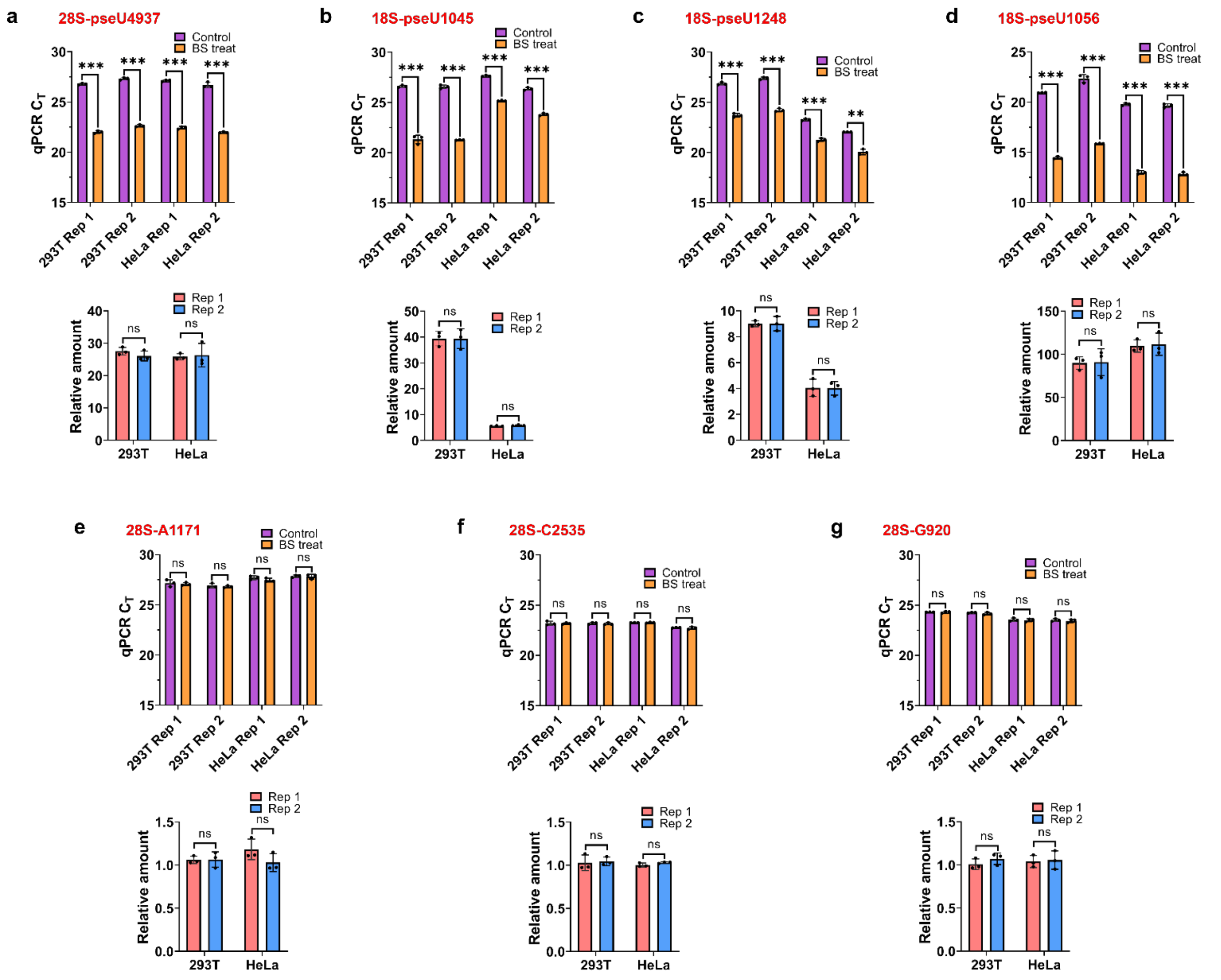

Figure S4. pseU-TRACE enabled the selective detection of Ψ in rRNA. (a-d) Real-time fluorescence amplification threshold cycle (C_T values) (top) and the relative amount (bottom) showed that pseU-TRACE could detect Ψ in 28S and 18S. (e-g) Real-time fluorescence amplification threshold cycle (C_T values) (top) and the relative amount (bottom) showed pseU-TRACE results of other nucleotides including A, C and G in 28S. Specific Ψ sites were marked on the top left of the graphs. Each experiment was replicated twice (Rep1 and Rep2). Error bars indicated mean±s.d. for 3 technical replicates. *p<0.05; **p<0.01; ***p<0.001; ns, non-significant by t-test (one-tailed).

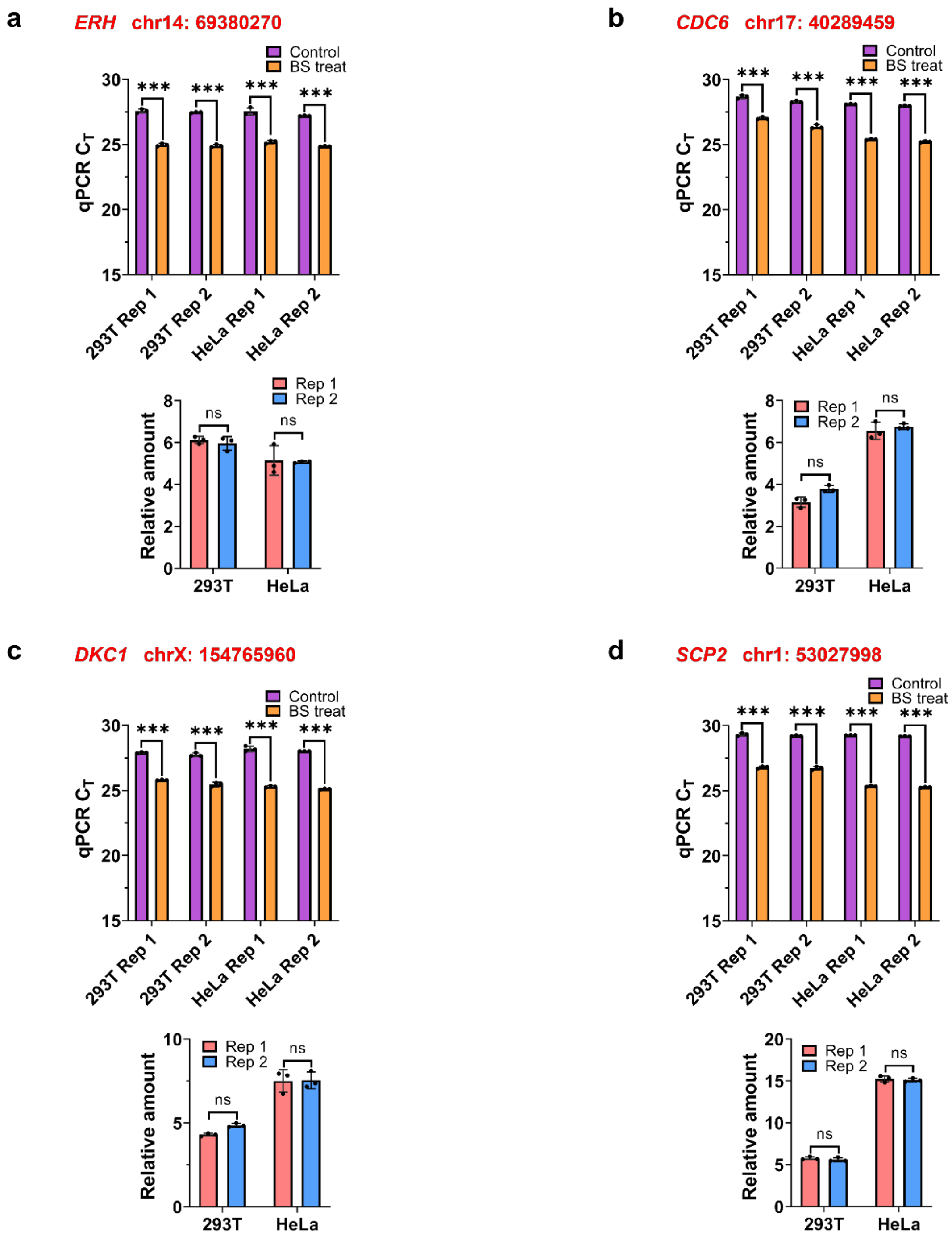

Figure S5. pseU-TRACE enabled the detection of Ψ in mRNA from total RNA samples. (a-d) Real-time fluorescence amplification threshold cycle (C_T values) (top) and the relative amount (bottom) demonstrated that pseU-TRACE detected Ψ in mRNA from HEK293T and HeLa total RNA. Specific Ψ sites were marked on the top left of the graphs. Each experiment was replicated twice (Rep1 and Rep2). Error bars indicated mean±s.d. for 3 technical replicates. *$p<0.05$; **$p<0.01$; ***$p<0.001$; ns, non-significant by t-test (one-tailed).

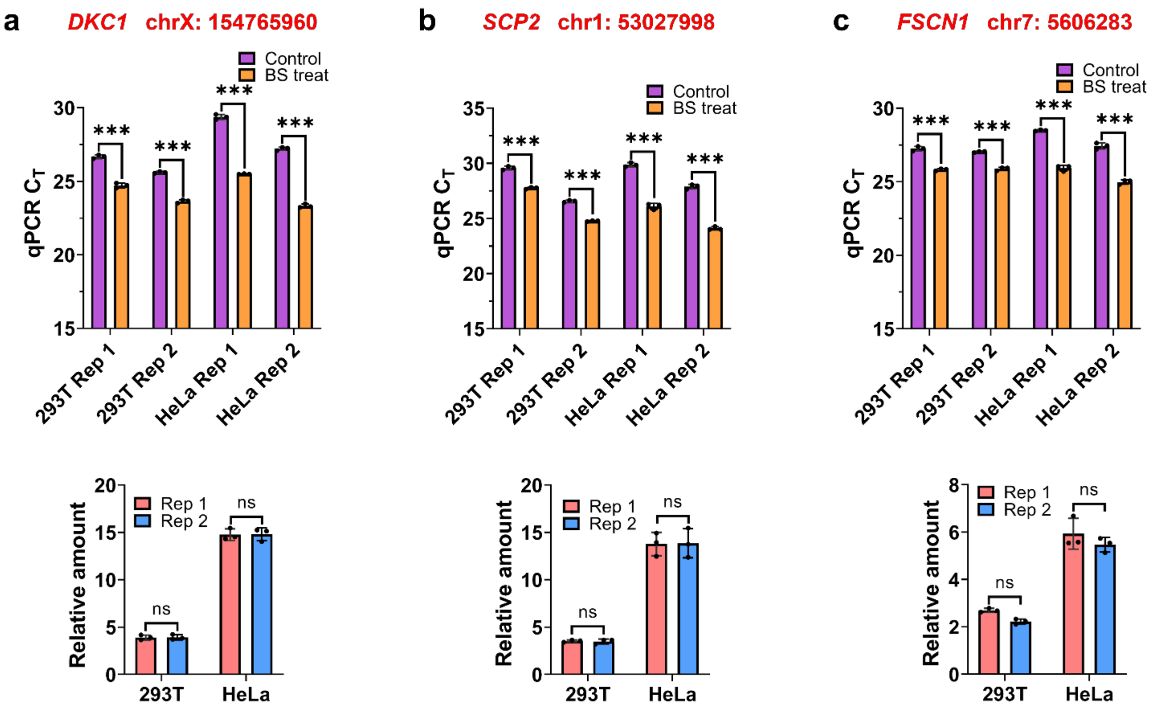

Figure S6. pseU-TRACE enabled the selective detection of Ψ in mRNA. (a-c) Real-time fluorescence amplification threshold cycle (C_T values) (top) and the relative amount (bottom) showed that pseU-TRACE could specifically identify Ψ in mRNA after Oligo(dT)$_{25}$ magnetic beads purification. Specific Ψ sites were marked on the top left of the graphs. Each experiment was replicated twice (Rep1 and Rep2). Error bars indicated mean±s.d. for 3 technical replicates. *p<0.05; **p<0.01; ***p<0.001; ns, non-significant by t-test (one-tailed).

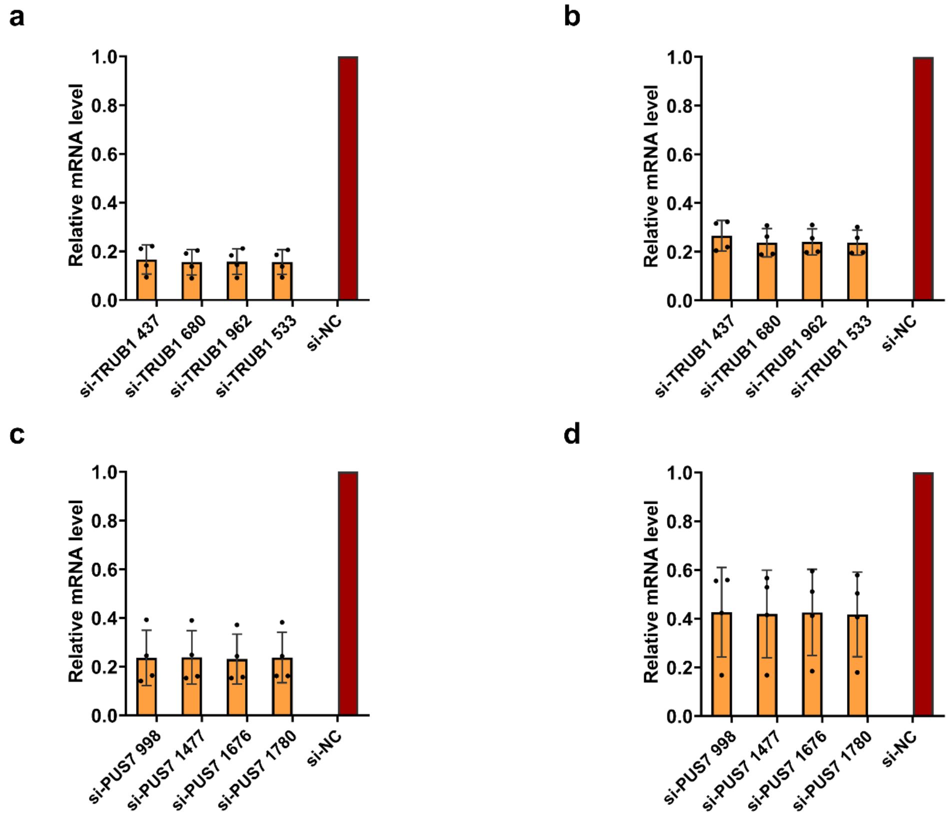

Figure S7. Detection of mRNA level after siRNA transfection. (a-b) The relative mRNA level was measured by qPCR after transfection of si-TRUB1 437, 680, 962, 533 or si-NC for 24 h (a) or 48 h (b). Using si-NC sample as a reference, the relative mRNA level was calculated by comparing with si-NC. (c-d) The relative mRNA level was measured by qPCR after transfection of si-PUS7 998, 1477, 1676, 1780 or si-NC for 24 h (c) or 48 h (d). Using si-NC sample as a reference, the relative mRNA level was calculated by comparing with si-NC. Error bars indicated mean±s.d. for 3 technical replicates.

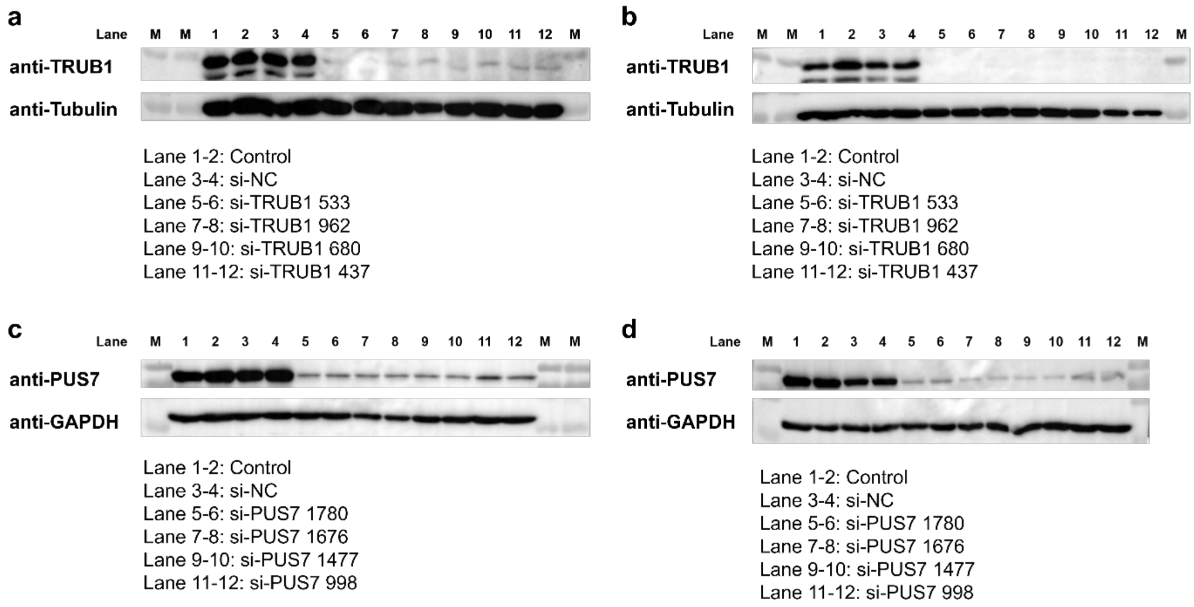

Figure S8. Detection of protein level after siRNA transfection. (a-b) Western blot showed that protein level was significantly reduced in samples transfected with si-TRUB1 437, 680, 962 and 533 compared to si-NC for 48 h (a) or 72 h (b). (c-d) Western blot showed that protein level was significantly reduced in samples transfected with si-PUS7 998, 1477, 1676 and 1789 compared to si-NC for 48 h (c) or 72 h (d). Two biological replicates were performed simultaneously.

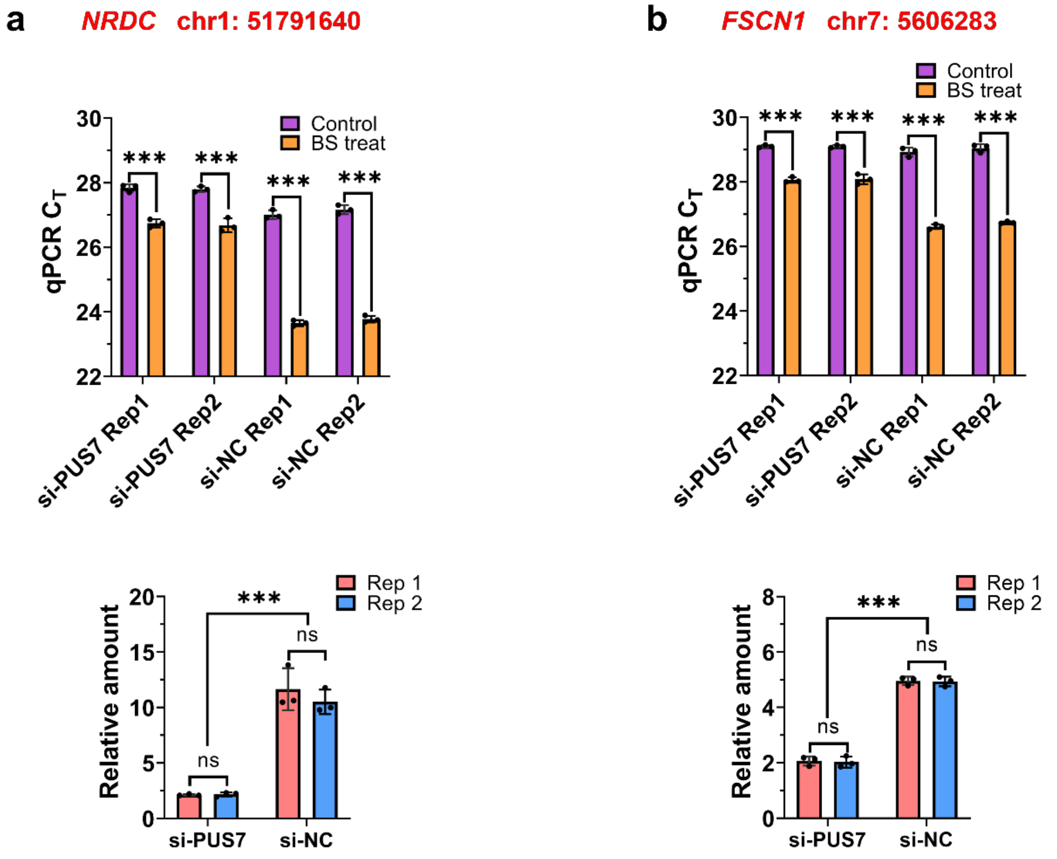

Figure S9. pseU-TRACE used for functional studies of Ψ regulation and metabolism. pseU-TRACE combined with siRNA interference used for the identification of Ψ under PUS7 regulation. (a-b) Real-time fluorescence amplification threshold cycle (C_T values) (top) and the amount (bottom) showed pseU-TRACE results for detecting Ψ in *NRDC* and *FSCN1* mRNA, comparing si-NC transfection with si-PUS7 1676 transfection. Specific Ψ sites were marked on the top left of the graphs. Each experiment was replicated twice (Rep1 and Rep2). Error bars indicated mean±s.d. for 3 technical replicates. *p<0.05; **p<0.01; ***p<0.001; ns, non-significant by t-test (one-tailed).

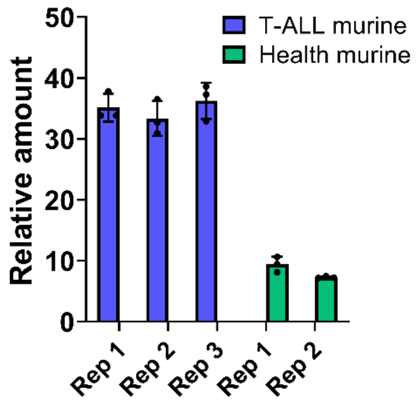

Figure S10. pseU-TRACE used for functional studies of Ψ associated with diseases. pseU-TRACE was employed to detect Ψ54 in U2 snoRNA in primary murine T-ALL and healthy murine thymocytes. Error bars indicated mean±s.d. for 3 technical replicates. *p<0.05; **p<0.01; ***p<0.001; ns, non-significant by t-test (one-tailed).

Table S1. Name and sequence of the RNA used in the experiment. The nucleotides marked in red were the selected detection sites.

Name	Sequence (5'-3')
19-Ψ	AAGUUGAAGΨGACAUCGCA
60-Ψ	CAGCACACGAAAACCAAGACΨACGCCGAAAGAGACACGCACGAACGAGACAGAACAGGAC
YW-U	CUUCGGUGUGGCUCUGCGUCGCUCAUGGGACUCACAGUAGCAGAGAGCACAACCGCCGAG
60-Ψ2	CAGCACACGAAAACCAAGACΨGCGCCGAAAGAGACACGCACGAACGAGACAGAACAGGAC
60-Ψ3	CAGCACACGAAAACCAAGAUΨUCGCCGAAAGAGACACGCACGAACGAGACAGAACAGGAC
28S-pseU3938 Ψ middle	GUGAAGAGACAUGAGAGGUGΨAGAAUAAGUGGGAGGCCCCCGGCGCCCCCCCGGUGUCCCC
28S-pseU3938 U middle	GUGAAGAGACAUGAGAGGUGUAGAAUAAGUGGGAGGCCCCCGGCGCCCCCCCGGUGUCCCC
mRNA SCP2 Ψ middle	AACUCAUCAAAUUGAAGCUGΨUCCAACCAGCUCUGCAAGUGAUGGAUUUAAGGCAAAUCUU
mRNA SCP2 U middle	AACUCAUCAAAUUGAAGCUGUUCCAACCAGCUCUGCAAGUGAUGGAUUUAAGGCAAAUCUU

Table S2. Name and sequence of the DNA for preliminary verification of pseU-TRACE used in the experiment. The nucleotide highlighted in red represented the difference between the two of DNAs.

Name	Sequence (5'-3')
59-T-G DNA	GTCCTGTTCTGTCTCGTTCGTGCGTGTCTCTTTCGGCGTGTCTTGGTTTTCGTGTGCTG
60-T-G DNA	GTCCTGTTCTGTCTCGTTCGTGCGTGTCTCTTTCGGCGTAGTCTTGGTTTTCGTGTGCTG
59-T-T DNA	GTCCTGTTCTGTCTCGTTCGTGCGTGTCTCTTTCGGCGTTTCTTGGTTTTCGTGTGCTG
60-T-T DNA	GTCCTGTTCTGTCTCGTTCGTGCGTGTCTCTTTCGGCGTATTCTTGGTTTTCGTGTGCTG
59-T-C DNA	GTCCTGTTCTGTCTCGTTCGTGCGTGTCTCTTTCGGCGTCTCTTGGTTTTCGTGTGCTG
60-T-C DNA	GTCCTGTTCTGTCTCGTTCGTGCGTGTCTCTTTCGGCGTACTCTTGGTTTTCGTGTGCTG
59-T-A DNA	GTCCTGTTCTGTCTCGTTCGTGCGTGTCTCTTTCGGCGTATCTTGGTTTTCGTGTGCTG
60-T-A DNA	GTCCTGTTCTGTCTCGTTCGTGCGTGTCTCTTTCGGCGTAATCTTGGTTTTCGTGTGCTG
59-G-G DNA	GTCCTGTTCTGTCTCGTTCGTGCGTGTCTCTTTCGGCGGGTCTTGGTTTTCGTGTGCTG
60-G-G DNA	GTCCTGTTCTGTCTCGTTCGTGCGTGTCTCTTTCGGCGGAGTCTTGGTTTTCGTGTGCTG
59-G-T DNA	GTCCTGTTCTGTCTCGTTCGTGCGTGTCTCTTTCGGCGGTTCTTGGTTTTCGTGTGCTG
60-G-T DNA	GTCCTGTTCTGTCTCGTTCGTGCGTGTCTCTTTCGGCGGATTCTTGGTTTTCGTGTGCTG
59-G-C DNA	GTCCTGTTCTGTCTCGTTCGTGCGTGTCTCTTTCGGCGGCTCTTGGTTTTCGTGTGCTG
60-G-C DNA	GTCCTGTTCTGTCTCGTTCGTGCGTGTCTCTTTCGGCGGACTCTTGGTTTTCGTGTGCTG
59-G-A DNA	GTCCTGTTCTGTCTCGTTCGTGCGTGTCTCTTTCGGCGGATCTTGGTTTTCGTGTGCTG
60-G-A DNA	GTCCTGTTCTGTCTCGTTCGTGCGTGTCTCTTTCGGCGGAATCTTGGTTTTCGTGTGCTG
59-A-G DNA	GTCCTGTTCTGTCTCGTTCGTGCGTGTCTCTTTCGGCGAGTCTTGGTTTTCGTGTGCTG

60-A-G DNA	GTCCTGTTCTGTCTCGTTCGTGCGTGTCTCTTTCGGCGAAGTCTTGGTTTTCGTGTGCTG
59-A-T DNA	GTCCTGTTCTGTCTCGTTCGTGCGTGTCTCTTTCGGCGATTCTTGGTTTTCGTGTGCTG
60-A-T DNA	GTCCTGTTCTGTCTCGTTCGTGCGTGTCTCTTTCGGCGAATTCTTGGTTTTCGTGTGCTG
59-A-C DNA	GTCCTGTTCTGTCTCGTTCGTGCGTGTCTCTTTCGGCGACTCTTGGTTTTCGTGTGCTG
60-A-C DNA	GTCCTGTTCTGTCTCGTTCGTGCGTGTCTCTTTCGGCGAACTCTTGGTTTTCGTGTGCTG
59-A-A DNA	GTCCTGTTCTGTCTCGTTCGTGCGTGTCTCTTTCGGCGAATCTTGGTTTTCGTGTGCTG
60-A-A DNA	GTCCTGTTCTGTCTCGTTCGTGCGTGTCTCTTTCGGCGAAATCTTGGTTTTCGTGTGCTG
59-C-G DNA	GTCCTGTTCTGTCTCGTTCGTGCGTGTCTCTTTCGGCGCGTCTTGGTTTTCGTGTGCTG
60-C-G DNA	GTCCTGTTCTGTCTCGTTCGTGCGTGTCTCTTTCGGCGCAGTCTTGGTTTTCGTGTGCTG
59-C-T DNA	GTCCTGTTCTGTCTCGTTCGTGCGTGTCTCTTTCGGCGCTTCTTGGTTTTCGTGTGCTG
60-C-T DNA	GTCCTGTTCTGTCTCGTTCGTGCGTGTCTCTTTCGGCGCATTCTTGGTTTTCGTGTGCTG
59-C-C DNA	GTCCTGTTCTGTCTCGTTCGTGCGTGTCTCTTTCGGCGCCTCTTGGTTTTCGTGTGCTG
60-C-C DNA	GTCCTGTTCTGTCTCGTTCGTGCGTGTCTCTTTCGGCGCACTCTTGGTTTTCGTGTGCTG
59-C-A DNA	GTCCTGTTCTGTCTCGTTCGTGCGTGTCTCTTTCGGCGCATCTTGGTTTTCGTGTGCTG
60-C-A DNA	GTCCTGTTCTGTCTCGTTCGTGCGTGTCTCTTTCGGCGCAATCTTGGTTTTCGTGTGCTG

Table S3. Name and sequence of the siRNA and si-NC used in the experiment.

Name	Sequence (5'-3')	
	Sense	Antisense
si-TRUB1 437	GGAGUUCUGGUUGUUGGAATT	UUCCAACAACCAGAACUCCTT

si-TRUB1 680	GGACAAAGACUUUCGACUUTT	AAGUCGAAAGUCUUUGUCCTT
si-TRUB1 962	GCACAGUCUCUUGAGCAUUTT	AAUGCUCAAGAGACUGUGCTT
si-TRUB1 533	GCUACUGAUACACUAGAUUTT	AAUCUAGUGUAUCAGUAGCTT
si-PUS7 998	GCUAGGGAAUUUCAGCUAUTT	AUAGCUGAAAUUCCCUAGCTT
si-PUS7 1477	GGAAUAACAUGGUAAGCAATT	UUGCUUACCAUGUUAUUCCTT
si-PUS7 1676	GCUCACAGCUGACAAUCUUTT	AAGAUUGUCAGCUGUGAGCTT
si-PUS7 1780	GCUGGGAAGUCGUUGCAUATT	UAUGCAACGACUUCCCAGCTT
si-NC	UUCUCCGAACGUGUCACGUTT	ACGUGACACGUUCGGAGAATT

Table S4. Name and sequence of the qPCR primers used in the experiment.

Name	Sequence (5'-3')
TRUB1-qPCR-FP	TCCCCTGTCCTTGAAACTGC
TRUB1-qPCR-RP	GCAGCTTCTCCTTCAACCGA
PUS7-qPCR-FP	CCATCAGTGAAGACGTGCCT
PUS7-qPCR-RP	GCCTCAGTGAGTCCATGCTT
GAPDH-qPCR-FP	TGCACCACCAACTGCTTAGC
GAPDH-qPCR-RP	GGCATGGACTGTGGTCATGAG
28S- qPCR- FP	GTGAAGAGACATGAGAGGTG
28S- qPCR- RP	GGGGACACCGGGGGGGCGCC

SCP2-qPCR-FP	AACTCATCAAATTGAAGCTG
SCP2-qPCR-RP	AAGATTTGCCTTAAATCCAT
pseU-TRACE-qPCR-FP	GATCTCGTATGCCGTCTTCTGCTTG
pseU-TRACE-qPCR-RP	AATGATACGGCGACCACCGAGATCT

Table S5. Name and sequence of the RT primers for reverse transcription used in the experiment.

Name	Sequence (5'-3')
GAPDH RT primer	GGCATGGACTGTGGTCATGAG
60-Ψ RT primer	GTCCTGTTCTGTCTCGTTCG
YW-U RT primer	CTCGGCGGTTGTGCTCTCTG
28S-pseU1569 RT primer	ATACCCAGGTCGGACGACCG
28S-pseU4937 RT primer	CTACGTACGAAACCCCGACC
28S-pseU3938 RT primer	GGGGACACCGGGGGGGCGCC
28S-U4897 RT primer	ATGGTTTAGCGCCAGGTTCC
28S-A1171 RT primer	CCTCCCCCGGGCCCGACGGC
28S-C2535 RT primer	GTCGCGTTACCGCACTGGAC
28S-G920 RT primer	CGACGGGTCTCGCTCCCTCG
18S-pseU1081 RT primer	CATGGGAATAACGCCGCCGC
18S-pseU1045 RT primer	GTTTATGGTCGGAACTACGA

18S-pseU1248 RT primer	ATCCTGTCCGTGTCCGGGCC
18S-pseU1056 RT primer	CGGTCGGCATCGTTTATGGT
18S-U1120 RT primer	ACCCAAAGACTTTGGTTTCC
5.8S- pseU69 RT primer	GTGCGTTCGAAGTGTCGATG
ERH-pseU RT primer	AGTATGTGAATGTTATAAAGTAGAT
AMFR-pseU RT primer	CTATAGCCCTGGACTTTCCC
CDC6-pseU RT primer	ATCTCCTGAAACAGCAGAGA
DKC1-pseU RT primer	TCCTGATATAGTCCCCAATC
SCP2-pseU RT primer	AAGATTTGCCTTAAATCCAT
NRDC-pseU RT primer	CCACAGTGACAGAAAATCCT
PTPRF-pseU RT primer	GGCCGGTTGTAGAAGCCCCG
FSCN1-pseU RT primer	GGAGGGTGCTGAGACGCCCC
U2-pseU RT primer	CTCCAAAAATCCATTTAATATATTGTCCTC

Table S6. Name and sequence of the probe primers for pseU-TRACE used in the experiment. The down primers had a phosphate modification at the 5' end.

Name	Sequence (5'-3')
59/60-T-G DNA up	AATGATACGGCGACCACCGAGATCTcagcacacgaaaaccaagac
59/60-T-G DNA down	acgccgaaagagacacgcacCAAGCAGAAGACGGCATACGAGATC
59/60-T-T DNA up	AATGATACGGCGACCACCGAGATCTcagcacacgaaaaccaagaa

59/60-T-T DNA down	acgccgaaagagacacgcacCAAGCAGAAGACGGCATACGAGATC
59/60-T-A DNA up	AATGATACGGCGACCACCGAGATCTcagcacacgaaaaccaagat
59/60-T-A DNA down	acgccgaaagagacacgcacCAAGCAGAAGACGGCATACGAGATC
59/60-T-C DNA up	AATGATACGGCGACCACCGAGATCTcagcacacgaaaaccaagag
59/60-T-C DNA down	acgccgaaagagacacgcacCAAGCAGAAGACGGCATACGAGATC
59/60-G-G DNA up	AATGATACGGCGACCACCGAGATCTcagcacacgaaaaccaagac
59/60-G-G DNA down	ccgccgaaagagacacgcacCAAGCAGAAGACGGCATACGAGATC
59/60-G-T DNA up	AATGATACGGCGACCACCGAGATCTcagcacacgaaaaccaagaa
59/60-G-T DNA down	ccgccgaaagagacacgcacCAAGCAGAAGACGGCATACGAGATC
59/60-G-A DNA up	AATGATACGGCGACCACCGAGATCTcagcacacgaaaaccaagat
59/60-G-A DNA down	ccgccgaaagagacacgcacCAAGCAGAAGACGGCATACGAGATC
59/60-G-C DNA up	AATGATACGGCGACCACCGAGATCTcagcacacgaaaaccaagag
59/60-G-C DNA down	ccgccgaaagagacacgcacCAAGCAGAAGACGGCATACGAGATC
59/60-A-G DNA up	AATGATACGGCGACCACCGAGATCTcagcacacgaaaaccaagac
59/60-A-G DNA down	tcgccgaaagagacacgcacCAAGCAGAAGACGGCATACGAGATC
59/60-A-T DNA up	AATGATACGGCGACCACCGAGATCTcagcacacgaaaaccaagaa
59/60-A-T DNA down	tcgccgaaagagacacgcacCAAGCAGAAGACGGCATACGAGATC
59/60-A-A DNA up	AATGATACGGCGACCACCGAGATCTcagcacacgaaaaccaagat

59/60-A-A DNA down	tcgccgaaagagacacgcacCAAGCAGAAGACGGCATACGAGATC
59/60-A-C DNA up	AATGATACGGCGACCACCGAGATCTcagcacacgaaaaccaagag
59/60-A-C DNA down	tcgccgaaagagacacgcacCAAGCAGAAGACGGCATACGAGATC
59/60-C-G DNA up	AATGATACGGCGACCACCGAGATCTcagcacacgaaaaccaagac
59/60-C-G DNA down	gcgccgaaagagacacgcacCAAGCAGAAGACGGCATACGAGATC
59/60-C-T DNA up	AATGATACGGCGACCACCGAGATCTcagcacacgaaaaccaagaa
59/60-C-T DNA down	gcgccgaaagagacacgcacCAAGCAGAAGACGGCATACGAGATC
59/60-C-A DNA up	AATGATACGGCGACCACCGAGATCTcagcacacgaaaaccaagat
59/60-C-A DNA down	gcgccgaaagagacacgcacCAAGCAGAAGACGGCATACGAGATC
59/60-C-C DNA up	AATGATACGGCGACCACCGAGATCTcagcacacgaaaaccaagag
59/60-C-C DNA down	gcgccgaaagagacacgcacCAAGCAGAAGACGGCATACGAGATC
60-Ψ up	AATGATACGGCGACCACCGAGATCTcagcacacgaaaaccaagac
60-Ψ down	acgccgaaagagacacgcacCAAGCAGAAGACGGCATACGAGATC
YW-U up	AATGATACGGCGACCACCGAGATCTgtgtggctctgcgtcgctca
YW-U down	gggactcacagtagcagagaCAAGCAGAAGACGGCATACGAGATC
60-Ψ up-A	AATGATACGGCGACCACCGAGATCTcagcacacgaaaaccaagaca
60-Ψ up-T	AATGATACGGCGACCACCGAGATCTcagcacacgaaaaccaagact
60-Ψ up-C	AATGATACGGCGACCACCGAGATCTcagcacacgaaaaccaagacc

60-Ψ up-G	AATGATACGGCGACCACCGAGATCTcagcacacgaaaaccaagacg
28S-pseU1569 up	AATGATACGGCGACCACCGAGATCTgaaactctggtggaggtccg
28S-pseU1569 down	agcggtcctgacgtgcaaatCAAGCAGAAGACGGCATACGAGATC
28S-pseU4937 up	AATGATACGGCGACCACCGAGATCTgggaacctggcgctaaacca
28S-pseU4937 down	tcgtagacgacctgcttctgCAAGCAGAAGACGGCATACGAGATC
28S-pseU3938 up	AATGATACGGCGACCACCGAGATCTgtgaagagacatgagaggtg
28S-pseU3938 down	agaataagtgggaggcccccCAAGCAGAAGACGGCATACGAGATC
28S-U4897 up	AATGATACGGCGACCACCGAGATCTggcggccgcccctcgcccg
28S-U4897 down	cacgcaccgcacgttcgtggCAAGCAGAAGACGGCATACGAGATC
28S-A1171 up	AATGATACGGCGACCACCGAGATCTgggcggggcggactgtcccc
28S-A1171 down	gtgcgccccgggcgggtcgcCAAGCAGAAGACGGCATACGAGATC
28S-C2535 up	AATGATACGGCGACCACCGAGATCTgatccccgaatccggagtgg
28S-C2535 down	ggagatgggcgccgcgaggcCAAGCAGAAGACGGCATACGAGATC
28S-G920 up	AATGATACGGCGACCACCGAGATCTttacagcccccccggcagca
28S-G920 down	cactcgccgaatcccggggcCAAGCAGAAGACGGCATACGAGATC
18S-pseU1081 up	AATGATACGGCGACCACCGAGATCTtaccgtcgtagttccgacca
18S-pseU1081 down	aaacgatgccgaccggcgatCAAGCAGAAGACGGCATACGAGATC
18S-pseU1045 up	AATGATACGGCGACCACCGAGATCTtcaagaacgaaagtcggagg

18S-pseU1045 down	tcgaagacgatcagataccgCAAGCAGAAGACGGCATACGAGATC
18S-pseU1248 up	AATGATACGGCGACCACCGAGATCTagcctgcggcttaatttgac
18S-pseU1248 down	caacacgggaaacctcacccCAAGCAGAAGACGGCATACGAGATC
18S-pseU1056 up	AATGATACGGCGACCACCGAGATCTagtcggaggttcgaagacga
18S-pseU1056 down	cagataccgtcgtagttccgCAAGCAGAAGACGGCATACGAGATC
18S-U1120 up	AATGATACGGCGACCACCGAGATCTatgcggcggcgttattccca
18S-U1120 down	gacccgccgggcagcttccgCAAGCAGAAGACGGCATACGAGATC
5.8S- pseU69 up	AATGATACGGCGACCACCGAGATCTgctagctgcgagaattaatg
5.8S- pseU69 down	gaattgcaggacacattgatCAAGCAGAAGACGGCATACGAGATC
ERH-pseU up	AATGATACGGCGACCACCGAGATCTCTtcgtcacttaatgttggt
ERH-pseU down	ccagtccttcaactgttcatCAAGCAGAAGACGGCATACGAGATC
AMFR-pseU up	AATGATACGGCGACCACCGAGATCTttggaaaaggaactcttggt
AMFR-pseU down	cgatacctggagcagaggagCAAGCAGAAGACGGCATACGAGATC
CDC6-pseU up	AATGATACGGCGACCACCGAGATCTggttctggacaatgctgcag
CDC6-pseU down	tcaattctgtgcccgcaaagCAAGCAGAAGACGGCATACGAGATC
DKC1-pseU up	AATGATACGGCGACCACCGAGATCTtatacacctcttgcatgtgg
DKC1-pseU down	tcaaatcctctgaagagagaCAAGCAGAAGACGGCATACGAGATC
SCP2-pseU up	AATGATACGGCGACCACCGAGATCTaactcatcaaattgaagctg

SCP2-pseU down	tccaaccagctctgcaagtgCAAGCAGAAGACGGCATACGAGATC
NRDC-pseU up	AATGATACGGCGACCACCGAGATCTtaccatgtctaccctacctg
NRDC-pseU down	aggaacacatccgggattctCAAGCAGAAGACGGCATACGAGATC
PTPRF-pseU up	AATGATACGGCGACCACCGAGATCTtgctcccggagacctttacc
PTPRF-pseU down	tgggggacaagaagaactacCAAGCAGAAGACGGCATACGAGATC
FSCN1-pseU up	AATGATACGGCGACCACCGAGATCTgtcccaggcaagcctggctg
FSCN1-pseU down	agtagcgagtgatctggcggCAAGCAGAAGACGGCATACGAGATC
U2-pseU up	AATGATACGGCGACCACCGAGATCTagtgtagtatctgttcttatcagt
U2-pseU down	taatatctgatacgtcctctatccCAAGCAGAAGACGGCATACGAGATC
